# A novel D-amino acid peptide with therapeutic potential (ISAD1) inhibits aggregation of neurotoxic disease-relevant mutant Tau and prevents Tau toxicity in vitro

**DOI:** 10.1186/s13195-022-00959-z

**Published:** 2022-01-21

**Authors:** Isabelle Aillaud, Senthilvelrajan Kaniyappan, Ram Reddy Chandupatla, Lisa Marie Ramirez, Sewar Alkhashrom, Jutta Eichler, Anselm H. C. Horn, Markus Zweckstetter, Eckhard Mandelkow, Heinrich Sticht, Susanne Aileen Funke

**Affiliations:** 1 Institute of Bioanalysis, Coburg University of Applied Sciences, Coburg, Germany; 2 German Center for Neurodegenerative Diseases (DZNE), Bonn, Germany; 3 Department of Neurodegenerative Diseases and Geriatric Psychiatry, University of Bonn, Bonn, Germany; 4 Forschungsgruppe Translationale Strukturbiologie, Deutsches Zentrum für Neurodegenerative Erkrankungen (DZNE), Göttingen, Germany; 5 Institut für Chemie und Pharmazie, Friedrich-Alexander-Universität Erlangen-Nürnberg, Erlangen, Germany; 6 Bioinformatik, Institut für Biochemie, Friedrich-Alexander-Universität Erlangen-Nürnberg, Erlangen, Germany; 7 Institut für Medizinische Genetik, Universität Zürich, Zürich, Switzerland; 8 Abteilung für NMR-basierte Strukturbiologie, Max-Planck-Institut für Biophysikalische Chemie, Göttingen, Germany; 9 CAESAR Research Center, Bonn, Germany

**Keywords:** Alzheimer’s disease, Tau aggregation inhibitors, Phage display, D-amino acid peptides, Therapy

## Abstract

**Background:**

Alzheimer’s disease (AD), the most common form of dementia, is a progressive neurodegenerative disorder that mainly affects older adults. One of the pathological hallmarks of AD is abnormally aggregated Tau protein that forms fibrillar deposits in the brain. In AD, Tau pathology correlates strongly with clinical symptoms, cognitive dysfunction, and neuronal death.

**Methods:**

We aimed to develop novel therapeutic D-amino acid peptides as Tau fibrillization inhibitors. It has been previously demonstrated that D-amino acid peptides are protease stable and less immunogenic than L-peptides, and these characteristics may render them suitable for in vivo applications. Using a phage display procedure against wild type full-length Tau (Tau^FL^), we selected a novel Tau binding L-peptide and synthesized its D-amino acid version ISAD1 and its retro inversed form, ISAD1rev, respectively.

**Results:**

While ISAD1rev inhibited Tau aggregation only moderately, ISAD1 bound to Tau in the aggregation-prone PHF6 region and inhibited fibrillization of Tau^FL^, disease-associated mutant full-length Tau (Tau^FLΔK^, Tau^FL-A152T^, Tau^FL-P301L^), and pro-aggregant repeat domain Tau mutant (Tau^RDΔK^). ISAD1 and ISAD1rev induced the formation of large high molecular weight Tau^FL^ and Tau^RDΔK^ oligomers that lack proper Thioflavin-positive β-sheet conformation even at lower concentrations. In silico modeling of ISAD1 Tau interaction at the PHF6 site revealed a binding mode similar to those known for other PHF6 binding peptides. Cell culture experiments demonstrated that ISAD1 and its inverse form are taken up by N2a-Tau^RDΔK^ cells efficiently and prevent cytotoxicity of externally added Tau fibrils as well as of internally expressed Tau^RDΔK^.

**Conclusions:**

ISAD1 and related peptides may be suitable for therapy development of AD by promoting off-pathway assembly of Tau, thus preventing its toxicity.

**Supplementary Information:**

The online version contains supplementary material available at 10.1186/s13195-022-00959-z.

## Introduction

Neurodegenerative diseases are often caused by protein misfolding resulting in the accumulation of protein deposits, such as amyloid fibrils [1]. Alzheimer disease (AD), the most common form of dementia, is characterized by two types of pathological protein deposits, extracellular amyloid plaques consisting of Amyloid-β (Aβ) peptide and intracellular neurofibrillary tangles (NFTs) consisting of Tau [2]. Tau is a microtubule (MT)-binding protein that promotes and stabilizes the assembly of MTs and the regulation of axonal transport [3, 4]. Binding of Tau to MT is regulated by post-translational modifications, especially by phosphorylation [5]. Tau phosphorylation negatively regulates the binding of Tau to MT; as a result, MT stabilization and axonal transport are compromised and Tau aggregates into insoluble fibrils.

The assembly of Tau protein into paired helical filaments (PHFs) depends on two short hexapeptide sequence motifs, _306_VQIVYK_311_ (PHF6) and _275_VQIINK_280_ (PHF6*), which are located at the beginning of the third and second repeat regions, respectively [6–9]. These motifs are important for filament assembly as they form a β-sheet structure [7, 10, 11]. Accordingly, the repeat domain (RD) of Tau is sufficient for forming PHFs which are thought to contribute to AD pathology [12–17]. In addition, there is evidence that Tau-induced neuronal toxicity is predominantly caused by smaller soluble oligomeric species formed in the Tau aggregation pathway rather than large protein deposits [18–22].

In recent years, a large number of potential therapeutic substances have been developed for the prevention of Aβ-based pathology in AD, such as Aβ production inhibitors, Aβ aggregation inhibitors, or Aβ antibodies [23–26]. Most of them failed in clinical trials due to side effects and lack of therapeutic success [27, 28]. Recently, only one Aβ drug candidate (aducanumab) obtained preliminary approval from the US Food and Drug Administration (FDA), while its effectiveness will still have to be proven [29–31]. In contrast to Aβ pathology, pathological changes in Tau correlate well with cognitive decline [32, 33]. A potential approach to developing Tau-directed therapies against dementia could involve targeting the beginning of the Tau fibrilization cascade, thereby preventing the formation of toxic oligomeric species which are hypothesized to propagate from cell to cell in a prion-like manner [22, 34].

A large number of Tau aggregation inhibitors have already been described as potential therapeutic agents [35–39]. In particular, D-amino acid peptides are emerging as promising drug candidates [40–42]. At least some D-peptides can be administered orally [43, 44] and are able to cross the blood-brain barrier in combination with high bioavailability [41, 43–48]. A promising D-peptide designated RD2, a derivate of D3 selected against D-Aβ-peptide using mirror image phage display, was shown to reduce plaque formation and inflammatory reactions and led to a significant improvement in the cognitive abilities of transgenic mice [43, 46–49]. The RD2 peptide has successfully completed phase 1 clinical trials. Several Tau-directed D-peptides have also been characterized in pre-clinical studies [8, 9, 50, 51]. While the D-peptides TLKIVW [9] and TD28 [50] were developed to bind PHF6, MMD3 [52] was selected against the hexapeptide sequence motif PHF6*.

In the present study, we selected a peptide against the wild type full-length Tau (Tau^FL^) protein in order to develop potential inhibitors acting on the early stages of a pathological fibrillization cascade. First, we selected a novel L-peptide ISAL1 using a phage display selection procedure with Tau^FL^ as a target and synthesized its D-amino acid counterpart, ISAD1, and its retro inversed version, ISAD1rev. We found that ISAD1 and its reversed form inhibit not only fibrillization of Tau^FL^, but also of several disease-associated mutant Tau variants. Furthermore, our novel D-peptides penetrate neuronal cells and prevent cytotoxicity induced by externally added pro-aggregant repeat domain Tau mutant ΔK280 (Tau^RDΔK^) fibril preparations as well as of internally expressed Tau^RDΔK^. Thus, our data suggest that our novel peptide ISAD1 has an improved potential for treatment of AD, whereas ISAD1rev inhibited Tau fibrillization only moderately.

## Materials and methods

### Tau protein expression and purification

The gene of the human Tau^FL^ isoform, encoding 441 amino acids (Tau 2N4R, Uniprot P10636-F), and the pro-aggregant mutant Tau^RDΔK^ were commercially synthesized and cloned into a pET28A(+) vector (Novagen, San Francisco, USA). Tau protein expression and purification was carried out according to Margittai et al. and Barghorn et al. with some modifications [53, 54]. The purity of the protein was analyzed by sodium dodecylsulfate polyacrylamide gel electrophoresis (SDS-PAGE). Protein concentrations were determined by the bicinchoninic acid (BCA) method.

### Preparation of Tau^FL^ fibrils for ELISA

The fibrillization was started by incubating 10 μM Tau^FL^ in 20 mM HEPES buffer, pH 6.8 with 2.5 μM heparin (16000 daltons (Da), H16K) at room temperature (RT) for 24 h. Fibril formation was verified using the Thioflavin-T (ThT) assay. For ThT fluorescence measurements, 20 μL of the sample with 10 μM ThT was pipetted into a black 96-well half area clear flat-bottom plate. Tau^FL^ without addition of heparin was used as a negative control. The fluorescence measurement was performed using a photometer POLARstar optima (BMG-Labtechnologies, Ortenberg, Germany), and excitation/emission wavelengths were set at 440/490 nm.

### Phage display selection

Selection of novel peptides binding to recombinant Tau^FL^ was performed by a phage display selection method. The target protein Tau^FL^ was prepared in 50 μg/ml concentration in coating buffer (0.1 M NaHCO_3_, pH 8.6) and immobilized on 96-well microtiter plates (Greiner Bio-One GmbH, Frickenhausen, Germany) overnight at 4 °C. The next day, the coating solution was discarded and each well was completely filled with blocking buffer (0.1 M NaHCO_3_, pH 8.6, 5 mg/ml bovine serum albumin (BSA)). After blocking for 1 h, the wells were washed 6 times with tris-buffered saline with Tween20 (TBST: TBS + 0.1 % [v/v] Tween-20). One hundred microliters of a 100-fold dilution of the phage library, displaying 12-mer random peptides (Ph.D.-12, New England Biolabs, Frankfurt a.M., Germany), was incubated for 1 h with agitation. To remove unbound phages, the wells were again washed 10 times with TBST. Bound phages were then eluted using 0.2 M Glycine-HCl (pH 2.2) with 1 mg/ml BSA. The phages were then amplified according to the manufacturer’s instructions (New England Biolabs, Frankfurt a.M., Germany) and used for the following 3 panning rounds.

### Single phage clone ELISA

A single clone binding assay was performed by enzyme-linked immunosorbent assay (ELISA) with the supernatant of amplified phage clones from selection round three and four to identify the single phages showing the strongest binding to Tau^FL^. Therefore, Tau^FL^ (50 μg/ml) diluted in coating buffer was immobilized on polystyrene 96-well microtiter plates (Greiner Bio-One GmbH, Frickenhausen, Germany) overnight at 4 °C. The control wells contained only buffer without target protein. The next day, the coating solution was discarded and incubated with 100 μl of blocking buffer (1 % BSA in 50 mM Tris, 150 mM NaCl, pH 7.6) for 1 h. To avoid the selection of possible BSA-binding phages, the supernatant of third and fourth selection round was mixed with blocking buffer in a ratio of 1:1 and pre-incubated for 20 min at RT with gentle agitation. After blocking, the plate was washed 6 times with TBST and 100 μl of the pre-incubated diluted samples was transferred into the appropriate wells. Subsequently, the plates were incubated for 1 h at RT with gentle agitation, followed by a washing step with TBST (6 times with 100 μl). Afterwards, 150 μl of the anti-M13 antibody dilution horseradish peroxidase (HRP)/anti-M13 (Monoclonal Conjugate; GE-Healthcare, Freiburg, Germany) was added to the adequate wells for 1 h at RT, followed by 6 times washing with TBST. The anti-M13 antibody was diluted 1:5000 in blocking buffer. Detection was conducted by measuring the conversion of the substrate tetramethylbenzidine (TMB) by HRP. One hundred microliters of the substrate solution was transferred to the respective sample wells. The enzyme reaction was stopped by adding 100 μl of 20 % [v/v] H_2_SO_4_. The absorption of the reaction product was measured at 450 nm in a Multiscan Go (Thermo Fisher Scientific, Darmstadt, Germany) microplate reader.

Positive phages from ELISA were selected for DNA isolation. DNA sequencing was performed at LGC Genomics (Berlin, Germany). The DNA sequences were translated into 12-mer amino acids, aligned using CLUSTAL Omega program (http://www.ebi.ac.uk/Tools/msa/clustalo/), and analyzed using the SAROTUP Database (an abbreviation of “Scanner And Reporter Of Target-Unrelated Peptides”) [55].

### Peptides

The selected peptide sequences obtained from the phage display selection were first synthesized as L-amino acid peptides. ISAL1 to 4 and ISAL9 (Table 1) were purchased from JPT Peptide Technologies (Berlin, Germany). L-peptides ISAL5 to ISAL8 and ISAL1sam were synthesized in the lab of Prof. Eichler as described in the supplement. Later, unlabeled and fluorescein amidites (FAM)-labeled peptides ISAD1 and ISAD1rev (all amino acids of both peptides are D-enantiomers) with > 95% purity were purchased from JPT Peptide Technologies as well. The FAM label is attached to the C-terminus of the peptides with an additional lysine residue in between. PHF6 and PHF6* were purchased as N-terminally acetylated hexapeptides to allow self-aggregation.

**Table 1:** Selected L-peptides from phage display selection against Tau^FL^

Nr.	Sequence	Frequency	Inhibition of Tau^FL^ aggregation	Name
1	SVFKLSLTDAAS	1/80	+	ISAL1
2	NHDMDLLVWWMN	1/80	+/-	ISAL2
3	NWSMPGMTQGFL	13/80	-	ISAL3
4	DFHQRDDDSQQA	1/80	-	ISAL4
5	AMYQFSRNPHLP	3/80	-	ISAL5
6	VSPAWDARTRSA	2/80	-	ISAL6
7	MTPHGNSKTPSG	1/80	-	ISAL7
8	HDWYRSPRMGLF	1/80	-	ISAL8
9	DLSHGQDLMHHH	1/80	-	ISAL9
10	SASVTSKFDALL	-	-	ISAL1sam

The peptide sequences were determined after DNA sequencing of the positive phages. Each sequence was given a number in the list. (+/-) indicates comparably low inhibition of Tau^FL^ fibrillization, (-) indicates that the peptide showed no effect on fibril formation. The scrambled peptide (ISAL1sam) was synthesized and did not show any inhibition of Tau^FL^ fibrillization

### Detection of peptide binding to Tau conformers using ELISA

A 96-well microtiter plate (Greiner Bio-One GmbH, Frickenhausen, Germany) was coated with 5 μg/ml Tau^FL^ or fibrils in coating buffer for incubation overnight at 4 °C. For investigation of the binding properties to the hexapeptide, 5 μM PHF6 was coated overnight. After three times washing with 300 μl phosphate-buffered saline with Tween20 (PBST: PBS with 0.1 % [v/v] Tween20), the plate was blocked with 3 % [w/v] BSA in PBS for 1 h at RT, followed by further washing steps. Subsequently, 100 μl FAM-labeled peptides was added at a final concentration of 0.1 to 20 μg/ml in PBST and incubated for 1 h at RT. The plate was washed three times with PBST before 100 μl sheep anti-fluorescein isothiocyanate (FITC) HRP-conjugate (1:5000 dilution in PBST; AbD Serotec, Puchheim, Germany) was added and incubated for 1 h at RT with gentle agitation. Again, the plate was washed for three times, followed by addition of the TMB substrate. The reaction was stopped with 20 % [v/v] H_2_SO_4_ and absorbance was measured at 450 nm.

### In silico modeling of binding mode of ISAD1 to PHF6 fibrils

Modeling of the ISAD1 peptide complex with PHF6 was guided by previous models of PHF6 with D-peptides TLKIVW [9] and TD28 [50]. ISAD1 was modeled in the same extended geometry and the same binding register as the TLKIVW and TD28 D-peptides. The binding register was chosen according to the position of a conserved Φ+Φ (Φ hydrophobic residue; + central positively charged residue) sequence motif present in all these PHF6-binding peptides. Modeling was performed with Sybyl 7.3 (Tripos Inc., St. Louis, USA) and UCSF Chimera [56]. Structural analysis of the complexes between the PHF6 oligomers and the docked peptides was performed with VMD [57].

### Fibrillization of Tau^FL^ monitored by ThT assay

Tau aggregation assays were performed under reducing conditions. Before the addition of heparin and peptides, a final concentration of 1 mM dithiothreitol (DTT) was added to the Tau protein solution and heated at 95 °C for 10 min. For Tau^FL^ inhibition assays, 5 μM Tau^FL^ were incubated in HEPES buffer (pH 6.7) in the presence of 1.25 μM heparin at 37 °C for 48 h with or without novel D-peptides (ISAD1 and ISAD1rev) at different concentrations (1 nM to 200 μM). Final concentration of 10 μM ThT was used for monitoring fibrillization. In case of the two hexapeptides, 5 μM PHF6 and 5 μM PHF6*, respectively, without addition of the aggregation inducer heparin (16000 Da) were used for monitoring the fibrillization process. The assays were performed with 50-μl sample volume per well in a 96-well half area microtiter plate (Greiner Bio-One GmbH, Frickenhausen, Germany). The fibrillization of Tau^FL^ was monitored by ThT, and the relative fluorescence intensity of ThT was read out at 440 excitation/521 emission nm in a BMG microplate reader (BMG Labtech, Ortenberg, Germany).

### Fibrillization of Tau^RDΔK^ and Tau mutants monitored by Thioflavin-S (ThS) assay

For Tau fibrillization inhibition assays, 10 μM Tau mutant protein (Tau^RDΔK^, Tau^FLΔK^, Tau^FL-A152T^, Tau^FL-P301L^) was incubated in BES buffer (N,N-bis(2-hydroxyethyl)-2-aminoethanesulfonic acid, pH 7) in the presence of 2.5 μM heparin at 37 °C for 24 h with and without novel D-peptides (ISAD1 and ISAD1rev) at different concentrations (1 nM to 200 μM). Final concentration of 20 μM ThS was used for monitoring fibrillization. The assay was performed with 40-μl sample volume per well in a 384-well microtiter plate (Thermo LabSystems, Dreieich, Germany). The fibrillization of Tau was monitored by ThS and the relative fluorescence intensity of ThS was read out at 440 excitation/521 emission nm in a Tecan micro titer plate reader (Tecan, Männedorf, Switzerland).

### Dynamic light scattering (DLS)

After the ThS assay (end time point, 24 h), the samples were used for DLS measurements. Twenty microliters of the sample was placed in a quartz batch cuvette (ZEN2112) and the measurement was performed at 25 °C in a Zetasizer Nano S instrument (Malvern Instruments, Herrenberg, Germany). The sample was thermally equilibrated at 25 °C for 2 min. The mean value of the intensities of an individual sample was determined over 3 measurements with 15 runs each. Analysis and averaging of the collected data were performed with the Zetasizer software 7.11 (Malvern Instruments, Herrenberg, Germany) and the result is represented as a volume graph. Tau^RDΔK^ fibrils (Tau+heparin) formed in the absence of D-peptides was used as a positive control.

### Pelleting assay and western blot

After the ThS assay (end time point, 24 h), 70 μl of each sample (pooled together from 2 wells) was centrifuged in a Beckmann coulter (Optima Max Ultra Centrifuge, TLA 100.3 rotor) at 61,000 rpm for 60 min at 4 °C. After centrifugation, the supernatant was separated from the pellet. Then, the pellet was dissolved in BES buffer in an equal volume as the supernatant. For the following western blot, 12-μl samples were mixed with 3-μl SDS-sample buffer (5x), heated for 5 min at 95 °C, and loaded onto a 8–16% SDS tris-glycine-gel (BioRad, Feldkirchen, Germany). The proteins were transferred to a polyvinylidene fluoride (PVDF) membrane. After the transfer, the membrane was blocked in 5% non-fat dry milk. After washing the membrane three times for 10 min with TBST, the primary pan-Tau K9JA antibody (1:5000; Agilent, Waldbronn, Germany) was incubated for 1 h at RT with gentle agitation, followed by again 3-times washing with TBST. For detection on western blot, the secondary antibody (goat anti-rabbit HRP, Agilent, Waldbronn, Germany) was incubated in a 1:2000 dilution for 1 h at RT with shaking. After another washing step (3 times with TBST), imaging was done with chemiluminescence substrate (Amersham^TM^, ECL Prime Western Blotting Detection Reagents, GE Healthcare, Chicago, USA) and Image Quant^TM^ LAS 4000 (GE Healthcare, Chicago, USA). The quantification of intensities was performed using ImageJ.

### Cell culture

Cells of a Neuro-2a (N2a) Tau^RDΔK^ inducible cell line (N2a-Tau^RDΔK^) [58] were grown in minimal essential media (MEM, Sigma Aldrich, Darmstadt, Germany) supplemented with 10% fetal bovine serum (FBS), 5 ml non-essential amino acids (PAA, Pasching, Austria), and 1X penicillin and streptomycin antibiotics at 37 °C with 5% CO_2_. The inducible N2a cell line expressing Tau^RDΔK^ require antibiotics geneticin G418 (300 μg/ml) and hygromycin (100 μg/ml). Tau^RDΔK^ expression was induced by incubating cells with 1 μg/ml doxycycline (Dox) in the studies on the detoxification of cellular Tau^RDΔK^ by the D-peptides; otherwise, these cells did not express the Tau^RDΔK^ protein.

### Cell viability assays

Cell viability was analyzed in accordance with the manufacturer’s protocol (Roche Diagnostics, Mannheim, Germany; cell proliferation kit II (MTT)). This assay is based on the cleavage of the yellow tetrazolium salt MTT into purple formazan dye by metabolic active cells. The color changes only in viable cells and can be directly quantified using a scanning multiwell spectrophotometer. In all experiments, the cells were grown as described previously. The cells (25,000 cells/well or 80% confluence) were plated on poly D-lysine-coated 96-well plates (Greiner Bio-One GmbH, Frickenhausen, Germany) for stronger attachment from overnight to 24 h at 37 °C. Fibrillized Tau^RDΔK^ was generated by incubating 200 μM Tau^RDΔK^ in BES buffer at 37 °C for 24 h in the presence of 2 mM D-peptides (Tau^RDΔK^:peptide = 1:10). Successful fibrillization was verified using ThS (5 μM Tau^RDΔK^, 10 μM ThS) measurement. The aggregated Tau^RDΔK^ (10 μM final concentration), Tau^RDΔK^ (10 μM) + peptide (100 μM) samples (ISAD1 and ISAD1rev), buffer only (negative control, set to 100% cell viability), and TritonX-100 (2%, cytotoxic agent, positive control) were incubated on N2a-Tau^RDΔK^ cells (100 μl solution) for another 24 h. The cell viability was measured in accordance with the manufacturer’s protocol.

### Measurement of lactate dehydrogenase (LDH) release

N2a cells expressing Tau^RDΔK^ were plated on poly D-lysine-coated 96-well plates with a density of 25,000 cells/well. At 70 to 80% confluence, the cells were treated for 24 h with various concentrations of D-peptides (25, 50, 100, and 250 μM) or Tau^RDΔK^ (10 μM final concentration) in the presence of ISAD1 and ISAD1rev (100 μM final concentration). The ability of D-peptides to neutralize the toxicity of Tau^RDΔK^ oligomers/fibrils was investigated by measuring the amount of released LDH (Roche Diagnostics, Mannheim, Germany). Therefore, 50 μL of each well was transferred to a fresh 96-well plate and 50 μL of reagent was added followed by a 30-min incubation period at RT. Finally, 50 μL of stop solution (1 N HCl) was added and absorbance was recorded at 492 nm (TECAN spectrofluorometer, Männedorf, Switzerland). Absorbance values were corrected by background values and the percentage of LDH release was calculated.

### Reactive oxygen species (ROS) measurements

Toxic Tau oligomers and fibrils can induce the production of superoxides and peroxy radicals in cells which can be measured with fluorescent dye dichlorodihydrofluorescein (DCF). N2a-Tau^RDΔK^ cells were plated on D-lysine-coated 96-well plates. At 70 to 80% confluence, the cells were washed once with warm PBS and then incubated with 20 μM of DCF (Abcam, Cambridge, UK) diluted in 1X dilution buffer for 30 min at 37 °C. After 30 min, the cells were washed once with 1X PBS. After washing, the cells were incubated with desired concentrations of different samples (10 μM oligomers/fibrils ± treated with 100 μM D-peptides or controls) for 30 min. The cytotoxic agent TBHP (tert-butylhydroperoxide) was used as the positive control. The fluorescence intensity was measured using a spectrofluorometer (Tecan, Männedorf, Switzerland; excitation at 485 nm and emission at 535 nm).

### Effect of ISAD1 and ISAD1rev on cellular Tau^RDΔK^ aggregation

After the N2a-Tau^RDΔK^ cells reached the desired confluence (25,000 cells/well or 80%), intracellular Tau^RDΔK^ expression was induced by the addition of 1 μg/ml Dox. The cells were plated on poly D-lysine-coated 96-well plates (Greiner Bio-One GmbH, Frickenhausen, Germany) and treated with ISAD1 and ISAD1rev in a concentration range of 25 to 250 μM. TritonX-100 (2%) was used as a positive control. The incubation and Tau^RDΔK^ expression time was 72 h. Following incubation, cell viability was studied by measuring MTT and LDH release in accordance with the manufacturer’s protocol.

## Results

### Phage display selection against Tau^FL^

Selection of peptides against recombinant Tau^FL^ was performed using a commercial peptide library of phages encoding > 1 × 10^9^ different random 12-amino acid sequences. Briefly, Tau^FL^ was immobilized on microtiter plates for the selection procedure. In case of the 441 amino acid Tau^FL^, a direct mirror image phage display selection to generate D-amino acid peptides was not practicable, as synthesis of Tau^FL^ consisting of D-amino acids was not possible. After four rounds of biopanning, single phages of the finally enriched phage dilution were tested for their ability to bind Tau^FL^ using single phage ELISA. The peptide sequences of promising phages, which showed relatively high signal in comparison to the negative control, were determined by DNA sequence analysis of the respective genome region. We found one single dominating amino acid sequence (consensus sequence) in 80 phages and some sequences were selected twice or three times (Table 1). All L-peptide sequences found after selection were compared to already known peptide sequences listed in the SAROTUP database (“Scanner And Reporter Of Target-Unrelated Peptides”) [55] to exclude possible target-unrelated peptides (TUPs) from biopanning results. Nine L-peptides were chosen for further characterization and for testing their potential to inhibit Tau^FL^ aggregation using the ThT fibrillization assay (Table 1). To our surprise, the L-peptides that carried the consensus sequence (ISAL3) did not inhibit Tau^FL^ aggregation. Instead, ISAL1 induced the highest reduction of Tau^FL^ fibrillization in the ThT assays.

Furthermore, to test whether ISAL1 inhibited Tau^FL^ fibrillization in a sequence-specific way, we performed additional ThT studies with a scrambled L-amino acid variant of ISAL1 (ISAL1sam), in which the amino acid sequence was randomly mixed. The scrambled control peptide did not inhibit the Tau^FL^ fibrillization (Sup. Fig. 1A) and showed no significant binding to Tau^FL^ (Sup. Fig. 1B) in ELISA.

As we intended to obtain Tau-binding-D-peptides for a later possible in vivo application, ISAL1 was synthesized as a D-peptide (ISAD1) and its retro inversed form (ISAD1rev) for further characterization.

### ISAD1 and ISAD1rev bind to both non-fibrillized Tau^FL^ and Tau^FL^ filaments

**Fig. 1:**
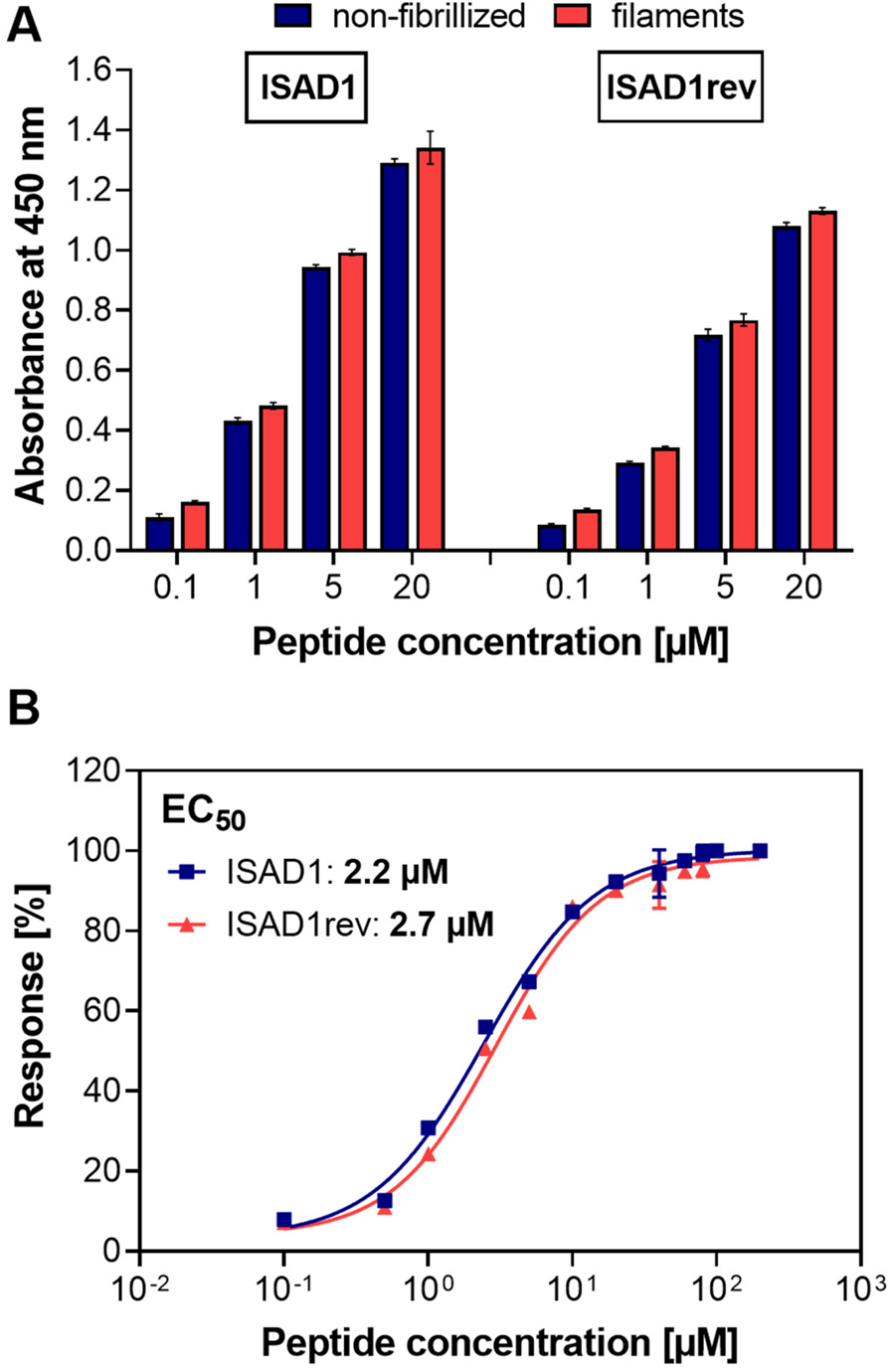
Binding properties of ISAD1 and ISAD1rev to non-fibrillized Tau^FL^ and Tau^FL^ filaments. **A** The plates coated with 5 μg/ml of Tau^FL^ and Tau^FL^ filaments were incubated with different concentrations of FAM-labeled peptides (ISAD1 and ISAD1rev) and the binding of the peptides was analyzed by ELISA. **B** Dose-response curve of Tau^FL^ with FAM-labeled peptides ISAD1 and ISAD1rev (0.1–200 μM) analyzed by ELISA. The EC_50_ was calculated for ISAD1 at 2.2 μM and ISAD1rev 2.7 μM, by non-linear regression

ELISA analysis demonstrated that ISAD1 and ISAD1rev bind to non-fibrillized Tau^FL^ and filaments of Tau^FL^ with similar strength (Fig. 1A). The half maximal effective concentrations (EC_50_) values of the peptides ISAD1 (2.2 μM) and ISAD1rev (2.7 μM) were calculated (Fig. 1B).

Furthermore, we investigated the interactions between ISAD1 and Tau^FL^ monomers by NMR which revealed only weak binding (Sup. Fig. 2A and 2B), similar to other previously identified peptides [9, 52]. However, binding of ISAD1 to unfibrilized Tau^FL^ was revealed by ELISA studies (Fig. 1A).

### In silico analysis of binding mode

**Fig. 2:**
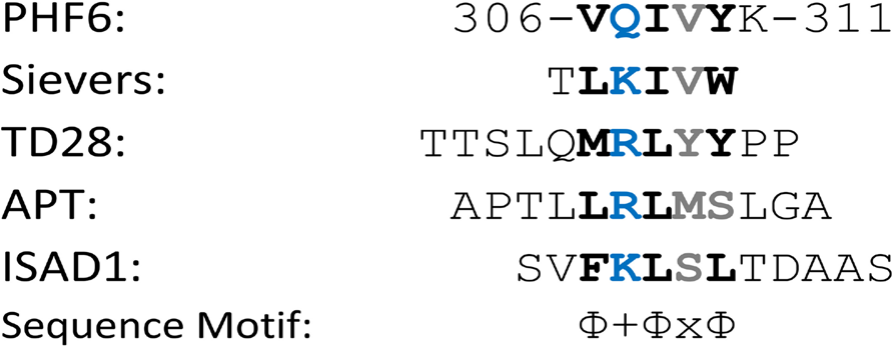
ISAD1 shows a similar binding motif to previously described PHF6 binding peptides. Sequence comparison of the ISAD1 peptide to PHF6 and other PHF6-binding peptides from the previous studies of Sievers et al. [9] and Dammers et al. [50]. The peptides are aligned according to the presence of a conserved basic (+) residue (shown in blue) which interacts with Q307 of PHF6 (shown in bold green). In all the peptides, this basic residue is flanked by two hydrophobic (Φ) residues (shown in bold) resulting in a Φ+Φ motif. In some peptides, an additional hydrophobic residue is present spaced by a variable (x) residue resulting in a Φ+ΦxΦ motif (shown in bold)

Using ELISA, the peptide PHF6 emerged as one possible binding site of ISAD1 (Sup. Fig. 3A). Subsequently, we compared the sequence of ISAD1 to several other D-peptides that were previously reported to bind to the PHF6 site of Tau (Fig. 2). As a common sequence motif, all these peptides exhibit a basic residue (Fig. 2: blue bold letter) flanked by two hydrophobic residues (Fig. 2; two bold black letters) resulting in a Φ+Φ motif. In some of the peptides (for example: ISAD1; TD28), an additional hydrophobic residue is present spaced by a variable residue resulting in a Φ+ΦxΦ motif (Fig. 2; all bold black letters). Notably, this sequence motif is absent in the PHF6*-binding peptides MMD3 and MMD3rev reported in Malhis et al. [52].

**Fig. 3:**
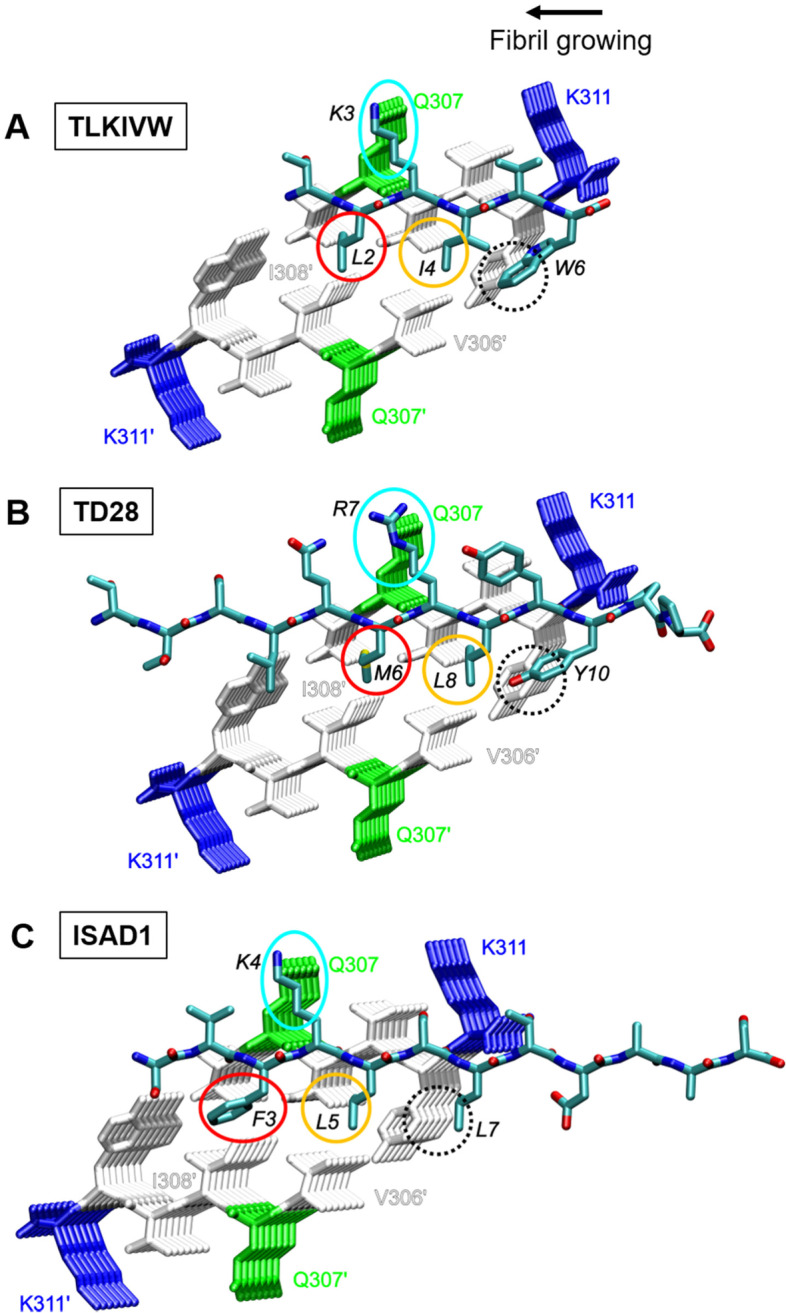
Model of the interaction of D-peptides with the PHF6 fibril residues. PHF6 fibrillar oligomer VQIVYK (PDB:2ON9 [10];) complexed with the following D-peptides: **A** TLKIVW [9], **B** TTSLQMRLYYPP (TD28) [50], and **C** SVFKLSLTDAAS (ISAD1). All molecules are depicted as sticks. PHF6 residues are colored according to their type (polar: green; basic: blue; hydrophobic: white). Atoms in peptide ligands are colored according to their chemical elements. Blue ellipses mark the interactions between the anchoring basic residue in the ligand and Q307 in PHF6. Red, orange, and dotted black circles indicate the Φ-positions of the D-peptide. Structurally equivalent positions of different peptides are encircled by the same color. The black circle marks a position present only in those peptides that exhibit the extended hydrophobic motif. Key residues in PHF6 and the D-peptides are labeled. Residue numbers with a prime indicate the second PHF6 layer, and black italic numbers refer to the D-peptide

The presence of a common sequence motif in the PHF6-binding D-peptides (Fig. 2) prompted us to model ISAD1 in the same binding mode as TLKIVW [9] and TD28 [50] to investigate the PHF6-peptide interactions. Figure 3 shows the similarity between the four D-peptide complexes with PHF6. Similar to TLKIVW and TD28 and ISAD1 can establish favorable side chain contacts between the central positively charged residue (R in TD28; K in TLKIVW and ISAD1) and Q307 of PHF6 (Fig. 3: blue circle), which presents an anchor for the β-sheet register. Additional stabilizing interactions arise from the hydrophobic residues flanking the central basic residue (Fig. 3: red and yellow circles). Furthermore, the three D-peptides TLKIVW, TD28, and ISAD1 possess a third hydrophobic residue corresponding to the C-terminal position of the Φ+ΦxΦ motif that forms interactions to the steric zipper near the edge of the fibril (Fig. 3: black dotted circle). Here, we found that ISAD1 has a similar sequence motif and binding mode as previously described peptides.

### ISAD1 and ISAD1rev inhibit the fibrillization of Tau^FL^ and Tau^RDΔK^

**Fig. 4:**
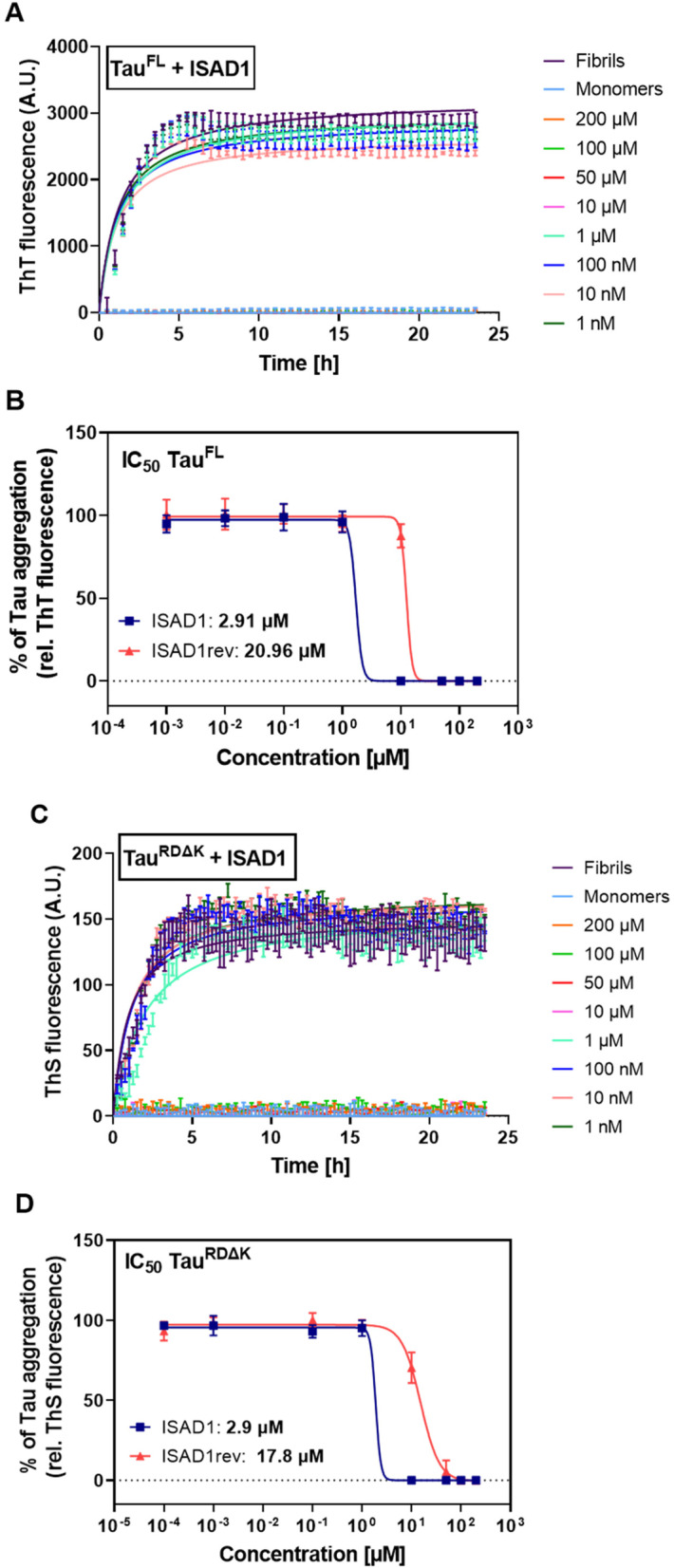
ISAD1 and ISAD1rev inhibit the aggregation of Tau^FL^ and Tau^RDΔK^. **A** Fibrillization curves of Tau^FL^ in the presence of different concentrations of the peptide ISAD1 over a period of 24 h. The assay was performed using 5 μM Tau^FL^ with 1.25 μM heparin (H16K) and 10 μM ThT in HEPES buffer. ISAD1 was added in concentrations of 0.1 nM to 200 μM. **B** Dose-response curve of Tau^FL^ fibrillization process in the presence of ISAD1 and ISAD1rev (0.1 nM–200 μM). **C** 10 μM Tau^RDΔK^ in BES buffer was incubated with 2.5 μM heparin and 20 μM ThS to monitor fibrillization. **D** Dose-response curve of Tau^RDΔK^ in the presence of ISAD1 and ISAD1rev. The relative fluorescence of a buffer sample was subtracted. Fluorescence was measured at 520 nm in relative units (mean ± standard deviations of results, three replicates per run). The fluorescence of Tau^FL^, Tau^RDΔK^ was set as 100% and the values and standard deviations of the other incubations are given as percentages of this maximum value. The data represent the mean values ± SD with *n* = 3 determinations

Initially, ISAD1 and ISAD1rev were tested for their potential to inhibit the heparin-induced fibrillization of Tau^FL^ and Tau^RDΔK^ using in vitro fibrillization assay monitored by ThT or ThS (Fig. 4). The formation of Tau^FL^ fibrils was monitored by ThT in the presence of different concentrations (1 nM–200 μM) of respective peptides. Fibrillization of Tau^FL^ was significantly reduced above 1 μM of peptide concentration (Fig. 4A). Based on the IC_50_ value, the ISAD1 peptide (2.91 μM) has a ~7 times higher efficiency in inhibiting fibrillization of Tau^FL^ than ISAD1rev (20.96 μM) (Fig. 4B).

Since the Tau^RD^ generally aggregates more efficiently than full-length Tau (where the other domains partially shield the repeat domain), we then tested the effects of the D-peptides on mutant Tau^RDΔK^ [6]. This again showed that ISAD1 is a much more potent aggregation inhibitor (IC_50_ – 2.9 μM) than its inversed version (IC_50_ – 17.8 μM) (Fig. 4C, D).

After identifying PHF6 as a binding site of ISAD1, we investigated the effect of ISAD1 on the fibrillization of PHF6 and PHF6* (Sup. Fig. 3B). At 25 μM concentration, ISAD1 inhibited fibril formation of PHF6, but not of PHF6*. None of the peptides showed self-fibrillization tendencies without Tau under assay procedure conditions (data not shown).

### ISAD1 and ISAD1rev inhibit the fibrillization of neurotoxic mutant Tau

**Fig. 5:**
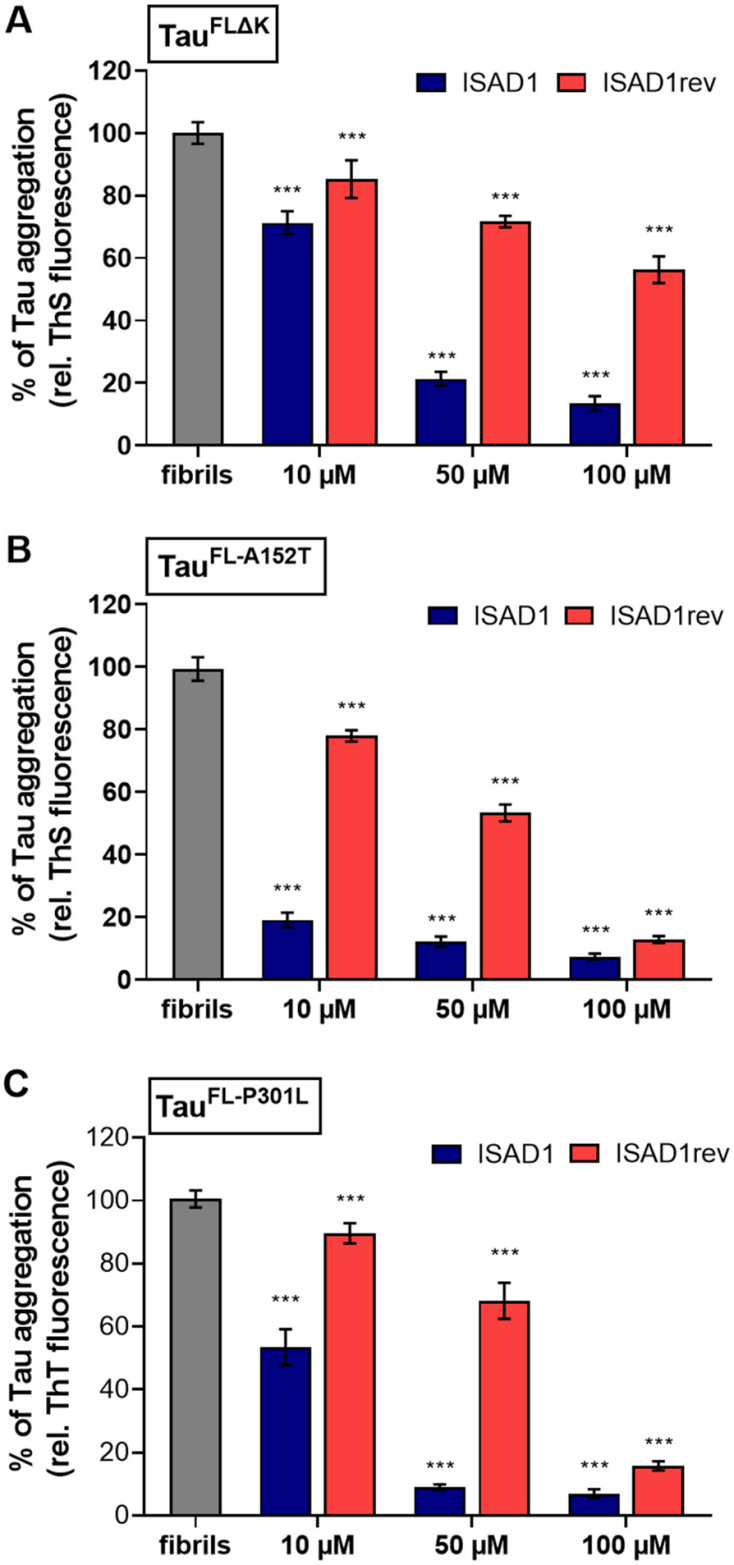
Inhibition of fibril formation of different Tau mutants in the presence of ISAD1 and ISAD1rev. For the fibrillization assay, 10 μM of each Tau mutant was diluted with 2.5 μM heparin and 20 μM ThS in BES buffer. The D-peptides ISAD1 and ISAD1rev were added to and incubated at 37 °C for 24 h. The fluorescence was measured every 30 min. The fluorescence of the respective Tau control after 24 h was set as 100%, and the values and standard deviations of the other incubations are given as percentages of this maximum value. **A** Inhibition of Tau^FLΔK^ fibrillization by ISAD1 peptides [*n* = 3; one-way ANOVA with Tukey’s post hoc test; *F*(6, 56) = 691; ***p ≤ 0.001]. **B** ISAD1 and ISAD1rev inhibit the fibrillization of Tau^FL-A152T^ [*n* = 3; one-way ANOVA with Tukey’s post hoc test; *F*(6, 56) = 2455; ***p ≤ 0.001]. **C** ISAD1 peptides show inhibition of Tau^FL-P301L^ fibrillization. Significant differences are shown with respect to the respective Tau isoform control (gray) [*n* = 3; one-way ANOVA with Tukey’s post hoc test; *F*(6, 56) = 809; ***p ≤ 0.001]

Next, we studied the effects of D-peptides in inhibiting the aggregation of physiologically relevant full-length Tau carrying disease-causing mutations such as ΔK280 (in R2 of RD), A152T (in proline-rich domain), and P301L (in R2 of RD) which are found in frontotemporal dementia with parkinsonism linked to chromosome 17 (FTDP-17), progressive supra-nuclear palsy (PSP), and AD patients. These mutations have only a mild effect on Tau-induced MT assembly but modulate the propensity for aggregation, which corresponds to toxic effects in transgenic mice [59–61]. In particular, mutations ΔK280 and P301S increase the propensity for β-structure of the repeat domain and thus strongly enhance fibrillization [17, 62], whereas mutation A152T (in the proline-rich domain P1) promotes oligomerization [63]. Therefore, we studied the ability of D-peptides to inhibit the fibrillization of three different Tau mutant forms (Tau^FLΔK^, Tau^FL-A152T^, Tau^FL-P301L^). ISAD1 inhibited the fibrillization of all these Tau mutants in a concentration-dependent manner (Tau:peptide = 1:1; 1:5; 1:10). At 10-μM concentration of D-peptide, ISAD1 already inhibited the fibrillization of A152T mutant Tau by 80% (Fig. 5B), and at 50 μM or more, we observed a complete inhibition (> 90%) of mutant Tau aggregation (Fig. 5A–C). ISAD1rev showed only weak inhibitory effects (Fig. 5A–C).

In summary, ISAD1 peptide strongly inhibited fiber growth approximately equally well, for Tau^FL^ or Tau^RDΔK^ repeat domain, with or without mutation. This is consistent with the model (Fig. 3) that the structures of the growing ends of fibers are similar, and similarly disrupted by the side chains on the inside of the two juxtaposed beta sheets.

### ISAD1 and ISAD1rev promote the formation of high molecular weight oligomers of Tau^RDΔK^

**Fig. 6:**
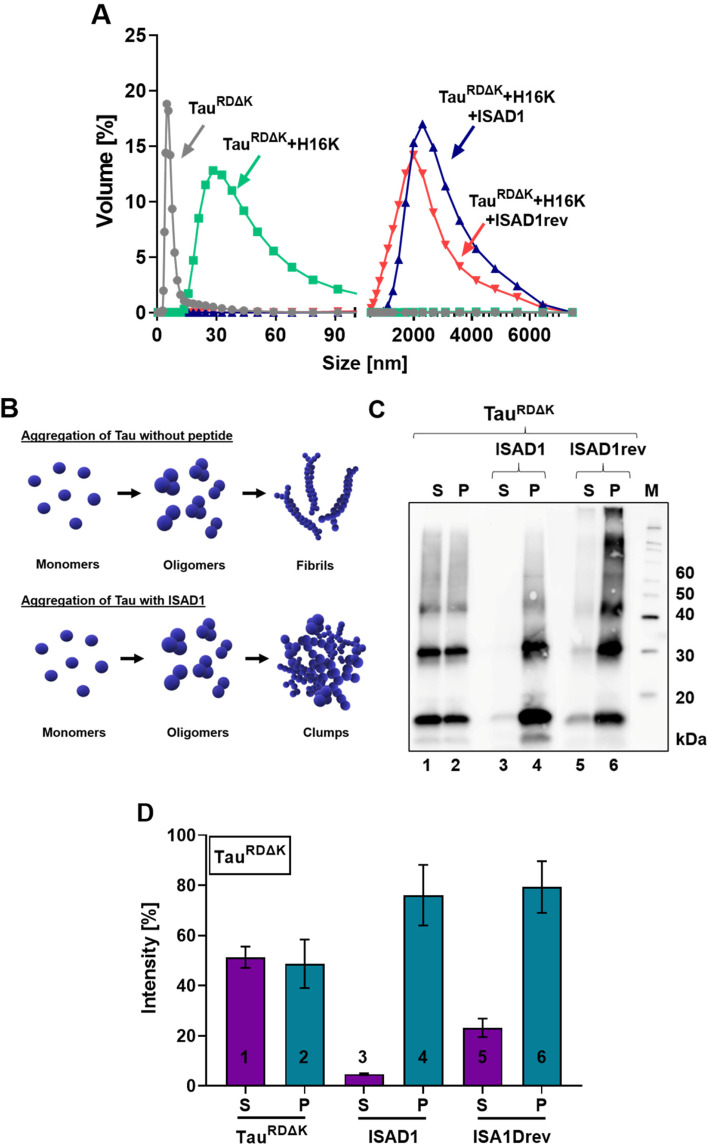
ISAD1 and ISAD1rev induce the formation of large Tau high-n oligomers, measured by DLS and pelleting assay. **A** Samples for DLS measurements were prepared with 10 μM Tau^RDΔK^, 2.5 μM heparin, and 20 μM ThS. The peptides were added in a concentration of 100 μM. All samples were incubated for 24 h. DLS was performed by 25 °C with an equilibration time of 2 min and 3 measurements within 15 runs. The average of the results is shown as a volume graph. Tau^RDΔK^ monomer shows a hydrodynamic size of < 10 nm (blue curve). When Tau is incubated with heparin, larger aggregates (PHF-like fibrils) with a size between 15 and 100 nm are formed (red curve). In the presence of ISAD1 and ISAD1rev, even larger high-n oligomers are formed, with a size of 1500–5000 nm (black and green curve), but without β-structure. **B** In general, fibrillization of monomeric Tau to fibrils is a multistep process that involves the formation of various aggregates, including protofibrillar oligomers. In the presence of ISAD1 peptides, Tau forms non-toxic clump-shaped high molecular oligomers. **C** Western blot of SDS-gels showing proteins in pellets (P) and supernatant (S) when 10 μM Tau was incubated with 100 μM peptide ISAD1 or ISAD1rev. After 24 h of Tau fibrillization, samples were centrifuged and the supernatant was separated from the pellet. The western blot was detected by the antibody K9JA. Tau^RDΔK^ fibrils resolved on SDS-gels show fractions in the supernatant (S) and pellet (P) after centrifugation (lanes 1,2). Due to the high molecular oligomers which are formed in the presence of the D-peptides, there are only apparent in the pellet (lanes 3–6). **D** The quantification of the western blot was performed using ImageJ. The intensity of protein amount in supernatant and pellet of the fibrils was set as 100%. There is a difference between the Tau fibrils and D-peptide-treated supernatant and pellet fractions

We next tested the nature of the non-fibrillar aggregates formed under the conditions described above by DLS and pelleting assay. As shown in Fig. 6A, in the absence of D-peptide, Tau^RDΔK^ forms aggregates in the size range of 30–90 nm. Tau monomers are in the size range of < 10 nm. In the presence of ISAD1 and ISAD1rev, the size of these aggregates significantly increased (~2000–6000 nm in size). In support of the DLS data, pelleting assay also demonstrated a higher amount of pelletable material in the presence of D-peptides than in the control samples (Fig. 6C, D). Similarly, all full-length disease-relevant Tau mutants (Tau^FLΔK^, Tau^FL-A152T^, Tau^FL-P301L^; Sup. Figs. 4–6) in the presence of ISAD1 formed high molecular weight oligomers with a size of 1000–8000 nm. Its inversed form did not induce the formation of high molecular weight oligomers of Tau mutants, as the formed aggregates were in the range of ~20–150 nm in size similar to heparin-induced fibrils (Sup. Figs. 4–6). The pelleting assays support the data from DLS confirming that ISAD1 induces high molecular weight oligomers presumably non-fibrillar as they are ThS negative (Fig. 6B) (Sup. Fig. 4B-C, Sup. Fig. 5B-C; Sup. Fig. 6B-C). As control, D-peptides alone in the presence of heparin did not form larger aggregates (Sup. Fig. 7).

### N2a-Tau^RDΔK^ cells actively internalize D-peptides

**Fig. 7:**
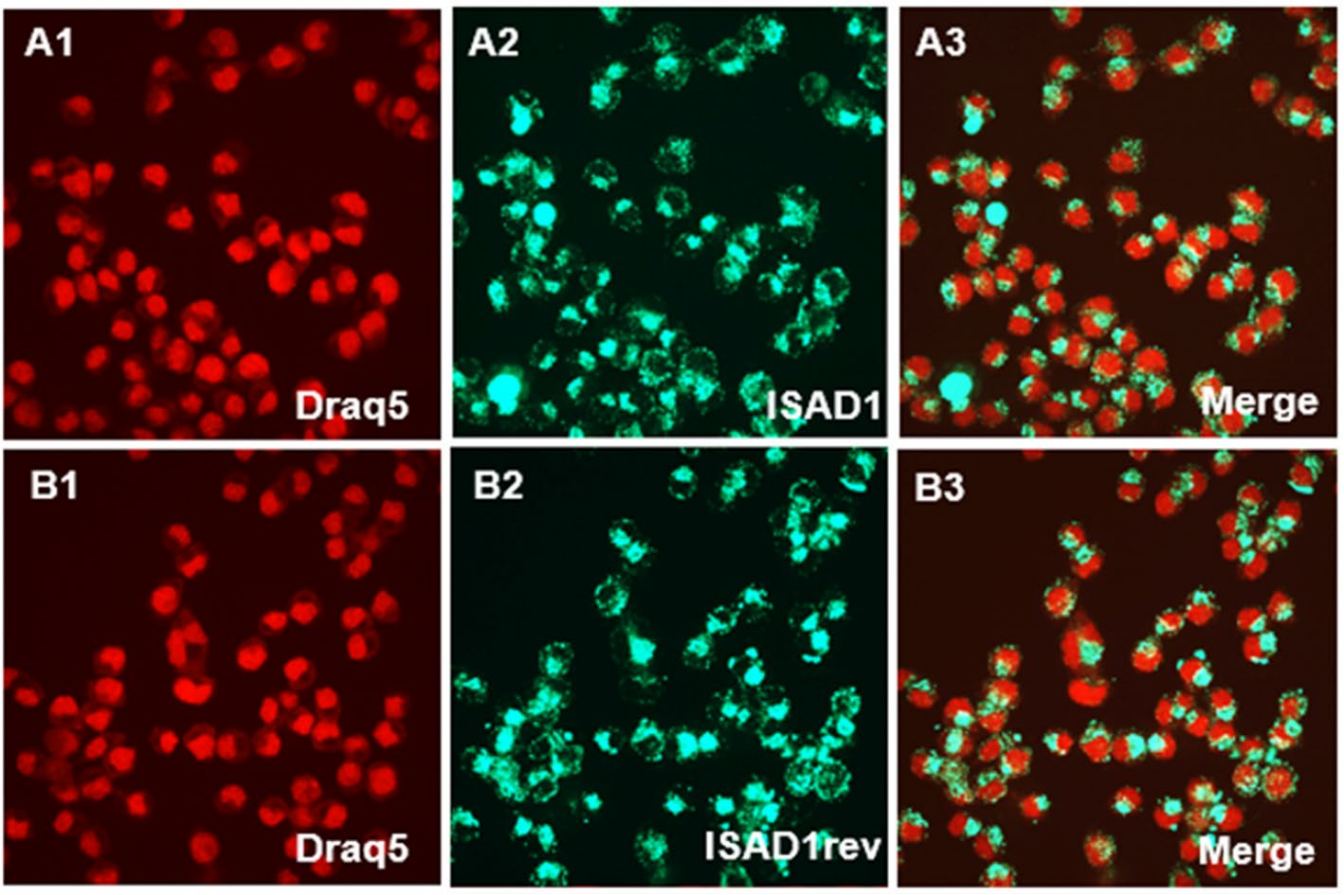
Uptake of fluorescence-labeled D-peptides analyzed by confocal microscopy. 25 μM FAM-labeled peptides (ISAD1 and ISArev) were incubated with N2a cells expressing Tau^RDΔK^ for 24 h. The uptake of D-peptides was analyzed by confocal microscopy. As shown in the representative images, the peptides are mainly localized in the cytoplasm (**A1**, **B1** Draq5 shown as red; **A2** ISAD1-FAM, **B2** ISAD1rev-FAM; **A3**, **B3** merged images)

In order to become potentially suitable for therapeutic development, the D-peptides should be internalized by neurons. To test this, 25 μM FAM-labeled ISAD1 or its inversed form was added to cells that express pro-aggregant mutant Tau^RDΔK^ for 24 h followed by cell fixation. The fixed cells were imaged by confocal microscopy, revealing that almost all cells took up the D-peptides (Fig. 7). Both ISAD1 and ISAD1rev accumulated in the cytosol but were excluded from the nucleus (Fig. 7: A3, B3).

### The D-peptides are not toxic to N2a-Tau^RDΔK^ cells even at elevated concentrations

**Fig. 8:**
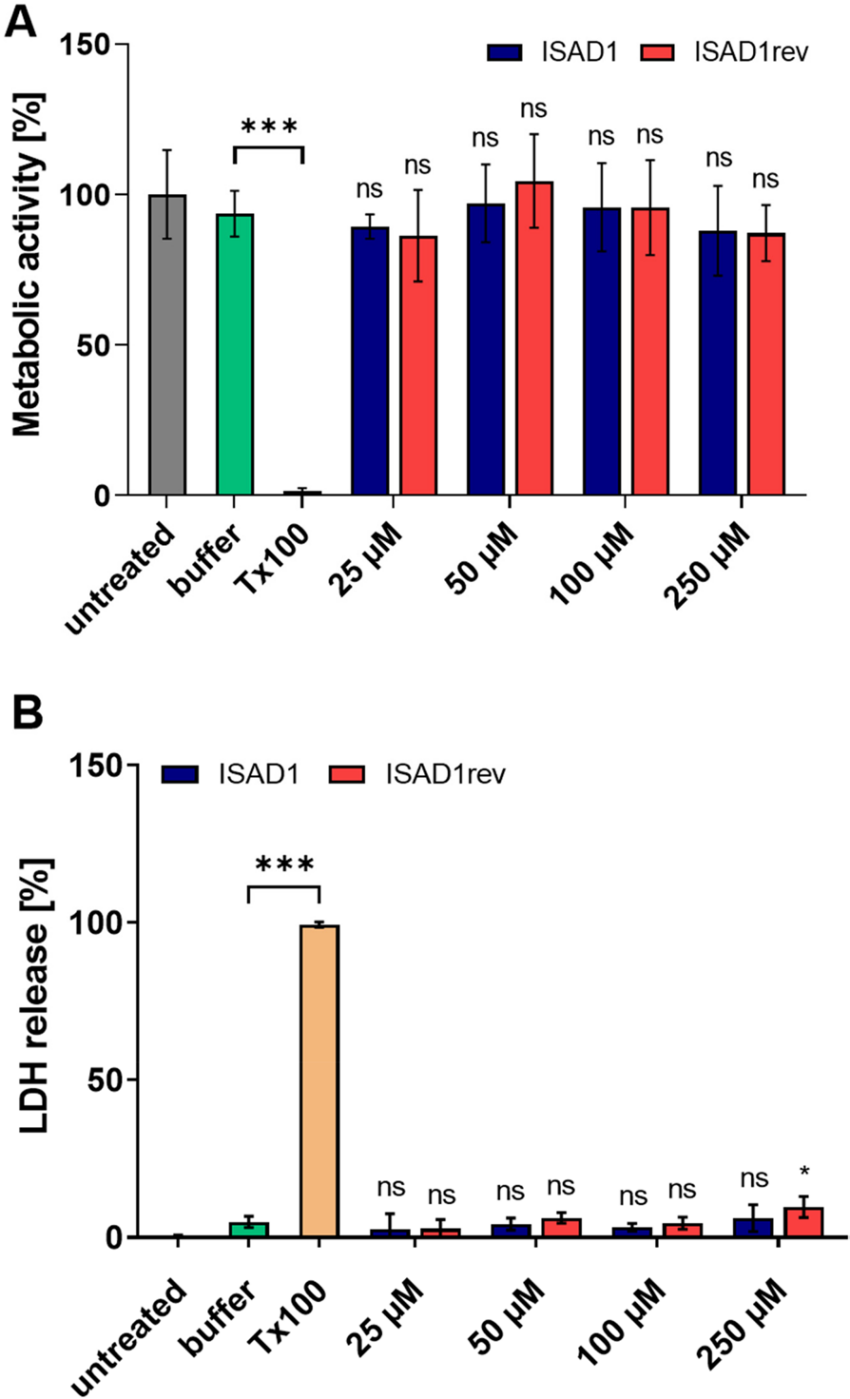
Peptides do not induce toxic effects in N2a-Tau^RDΔK^ cells. **A** MTT-based cytotoxicity assay in the presence of ISAD1 and ISAD1rev peptides. Different peptide concentrations (25, 50, 100, 250 μM) were tested on N2a-Tau^RDΔK^ cells over 24 h [*n* = 3; one-way ANOVA with Tukey’s post hoc test; *F*(10, 151) = 101; ***p ≤ 0.001]. **B** LDH release of N2a-Tau^RDΔK^ cells treated with ISAD1 peptides for 24 h [*n* = 3; one-way ANOVA with Tukey’s post hoc test; *F*(10, 88) = 1050; *p ≤ 0.05; ***p ≤ 0.001].

To find out whether the peptides cause toxic effects in N2a-Tau^RDΔK^ cells, we treated the cells with different concentrations of ISAD1 and its inversed version for 24 h. The cell viability and membrane integrity were tested by MTT and LDH assays, respectively. Figure 8A and B show that the peptides have no effect on these parameters even at higher concentrations up to 250 μM.

### The ISAD1 and ISAD1rev peptides prevent cell toxicity induced by Tau aggregates

**Fig. 9:**
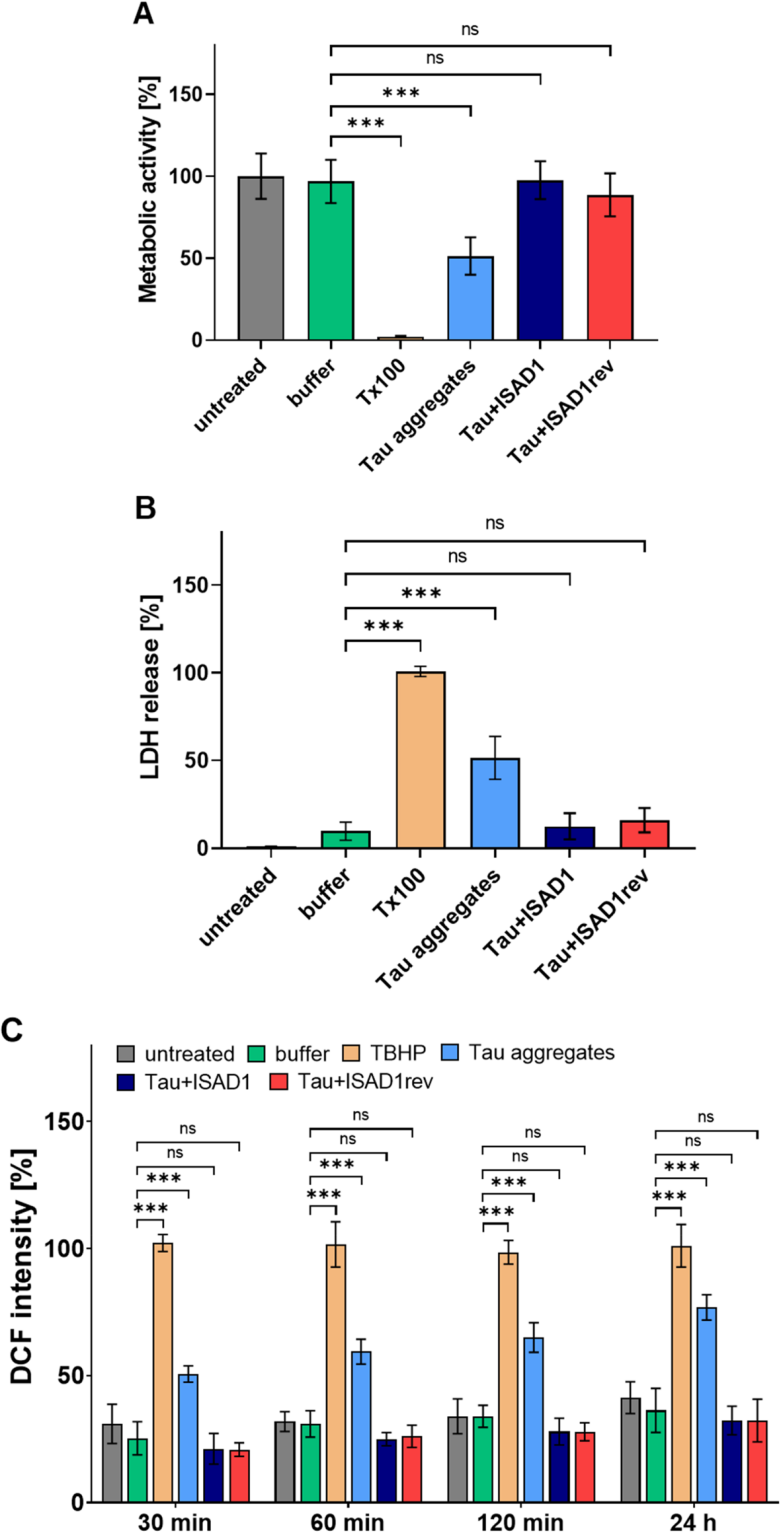
ISAD1 and ISAD1rev prevent toxic effects of Tau aggregates. 200 μM of Tau^RDΔK^ was incubated with or without peptides (1:10) respectively for 24 h at 37 °C. Pre-incubated samples (10 μM Tau, 100 μM peptide final concentration) were added to N2a-Tau^RDΔK^ cells followed by recording of MTT, LDH, and ROS intensities. **A** Pre-incubated Tau with peptides inhibit Tau toxic effects by preserving cell viability (blue and red bars) [*n* = 3; one-way ANOVA with Tukey’s post hoc test; *F*(5, 48) = 102; ***p ≤ 0.001]. **B** Low LDH release levels show protection of cell membrane integrity by peptides (blue and red bars) [*n* = 3; one-way ANOVA with Tukey’s post hoc test; *F*(5, 48) = 268; ***p ≤ 0.001]. **C** Within 30 min, the cells increase ROS level in the aggregate-treated cells (light blue bar). Pre-incubated Tau with peptides inhibit Tau toxic effects in reducing intracellular ROS levels (blue and red bars) compared to positive control TBHP (beige bar) [*n* = 3; one-way ANOVA with Tukey’s post hoc test; *F*(23, 192) = 209; ***p ≤ 0.001]

Tau aggregates in their fibrillar form (PHFs) are toxic to cells when treated extracellularly and cause reactive oxygen species production [21, 22, 34]. Therefore, we tested whether high molecular weight Tau oligomers formed in the presence of D-peptides still have the toxic effects of Tau, as judged by MTT, LDH, and ROS assays. We observed that Tau aggregates formed in the absence of D-peptides caused a reduction in the cell viability (Fig. 9A; light blue bar), compromised cell membrane integrity (Fig. 9B; light blue bar), and increased ROS production (Fig. 9C; light blue bar). Tau high molecular weight oligomers formed in the presence of ISAD1 (dark blue bar) or its inversed form (red bar) do not induce toxicity, as we observed no significant compromise in cell viability (Fig. 9A), cell membrane integrity (Fig. 9B), and ROS production (Fig. 9C) compared to respective controls.

**Fig. 10:**
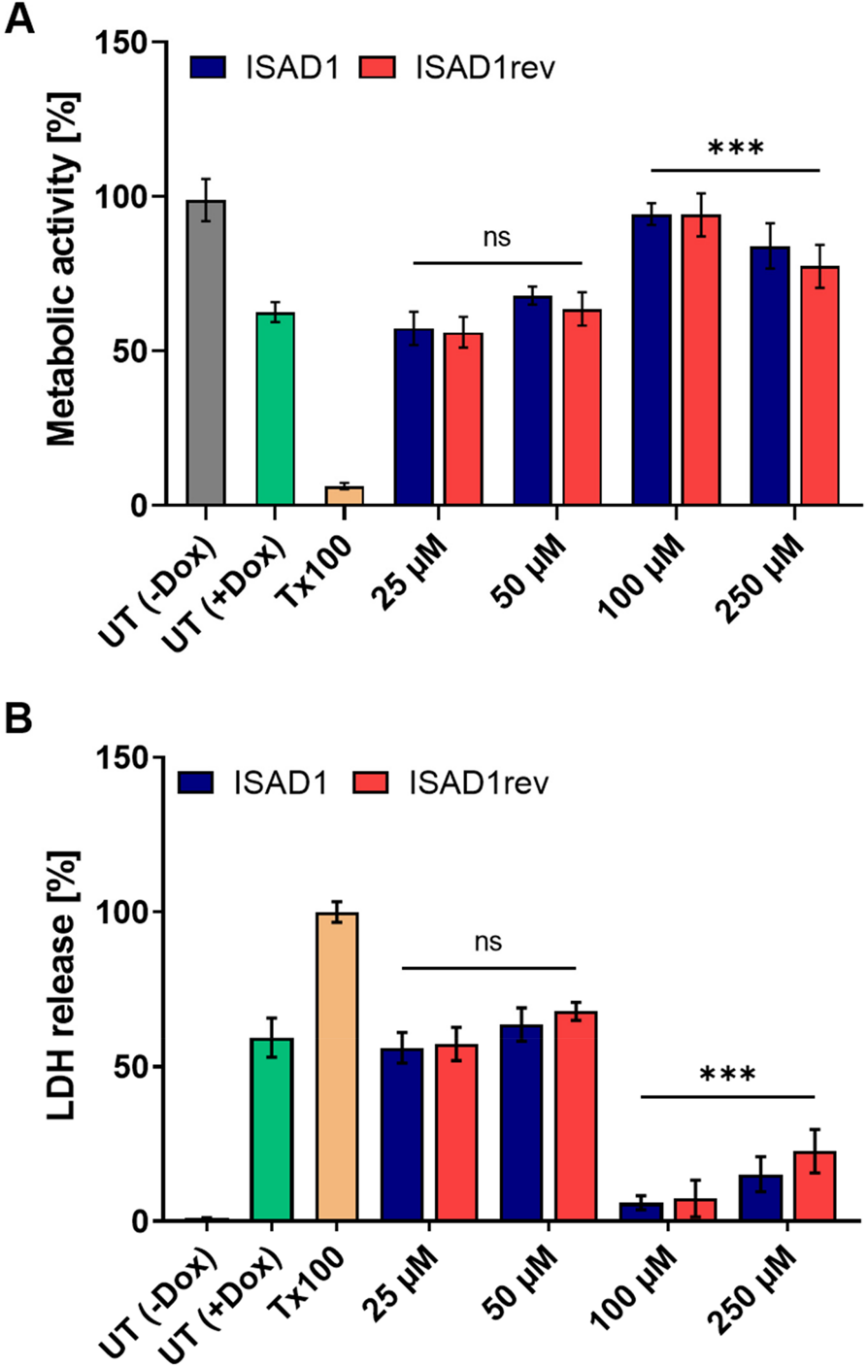
ISAD1 and ISAD1rev reduce toxic effects of intracellularly expressed Tau^RDΔK^ aggregates. Expression of Tau^RDΔK^ in N2a cells was induced by adding 1 μg/ml doxycycline. The cells were incubated with different concentrations of ISAD1 and ISAD1rev (25, 50, 100, 250 μM) for 24 h at 37 °C followed by determination of MTT and LDH intensities. **A** Peptides prevent intracellular toxic Tau effects by preserving metabolic activity from 100 μM concentration (blue and red bars) similar to untreated cells (gray bar, UT -Dox) [*n* = 2; one-way ANOVA with Tukey’s post hoc test; *F*(11, 60) = 126; ***p ≤ 0.001]. **B** Protection of cell membrane integrity by D-peptides (blue and red bars) determined by LDH release [*n* = 2; one-way ANOVA with Tukey’s post hoc test; *F*(11, 60) = 256; ***p ≤ 0.001].

To investigate the effect of D-peptides on inducible N2a cells expressing Tau^RDΔK^ (N2a-Tau^RDΔK^), we incubated the cells with the respective D-peptides in the presence of Dox and quantified the cell viability by MTT and LDH assays (Fig. 10). The metabolic activity of the compound-untreated control (gray bar) was set to 100%. The bar diagrams show the relative metabolic activity (Fig. 10A) and LDH release (Fig. 10B) of cells when treated with increasing amounts of ISAD1 and ISAD1rev for 72 h. After incubation with D-peptides, there was an increase in cell viability starting from 100 μM concentration, whereas treatment with 50 μM shows no increase in cell viability.

Taken together, the results show that the D-peptides are highly tolerable by cells and prevent Tau-induced toxicity by promoting Tau to form non-fibrillar high-n oligomers rather than amyloid-like fibers.

## Discussion

Even through Alois Alzheimer characterized AD more than 100 years ago, there is currently no curative treatment available*.* These circumstances and the predicted steady increase in the number of AD patients highlight the urgency of developing a causal AD therapy. Recently, the Aβ antibody aducanumab obtained preliminary approval from the FDA. Nevertheless, the approval is controversial and the efficacy of aducanumab has to be proven in further studies [29–31]. A number of potential therapeutic substances targeting the Aβ pathology appeared successful in pre-clinical models, but failed in clinical trials [64–66].

Apart from the amyloid peptide Aβ, another compelling target for AD therapy is Tau. A large body of evidence suggests that Tau pathology has a strong correlation with the disease progression and clinical symptoms [32], which has led to increased research into Tau-associated compounds [35, 67, 68]. Tau pathology not only occurs in AD but also in other neurological diseases, called tauopathies [69, 70]. The use of small D-amino acid peptides to prevent the pathological fibrillization of Tau may provide an alternative to small molecule non-peptide compounds [71, 72]. D-peptides are proven to be protease stable and less immunogenic than L-peptides which make them suitable for in vivo applications. The novel D-amino acid peptides we described here inhibited the aggregation not only of Tau^FL^, but also of disease-relevant Tau mutants.

ISAD1 and its synthesized reversed form show binding to both non-fibrillized Tau^FL^ and filaments of Tau^FL^ in ELISA studies. Both D-peptides bind to Tau^FL^ filaments with an estimated EC_50_ in the low micromolar range (Fig. 1). We found that ISAD1 binds to the PHF6 motif of Tau (Sup. Fig. 3A) and inhibits the fibrillization of PHF6, but not of PHF6* despite the close vicinity and high sequence similarity of these two hexapeptides. PHF6 (_306_VQIVYK_311_) is known to strongly promote Tau aggregation into β-structured filaments [6, 7], while most other parts Tau protein are unstructured. This could be the reason why phages in the selection process bind more preferentially to this structured sequence motif.

We illustrated the binding mode of ISAD1 to PHF6 fibrils by in silico modeling according to previously described Tau aggregation inhibitor peptides [9, 50] selected against PHF6. The modeling data indicated a similar sequence motif and binding mode in blocking PHF6 fibrillization as previously described PHF6-addressing D-peptides TLKIVW [9] and TD28 [50]. The modeling demonstrated the similarity between the three D-peptide complexes with PHF6. The formation of stabilizing backbone hydrogen bonds to PHF6 is allowed by binding in parallel β-sheet conformation. Since the interface is formed between a D- and L-peptide, a rippled β-sheet is created [73] which was shown by quantum chemistry calculations to exhibit favorable interaction energies in the PHF6 system [74]. A second effect, which blocks lateral fibril growth, results from the steric repulsion between the D-peptides and the second stack of β-strands (Fig. 3). It has been suggested that such steric repulsion between L2 in TLK or M6 in TD28, respectively, and V306’/I308’ of the second stack of β-strands represents key feature blocking further fibril growth [9, 50]. For ISAD1, the corresponding residue F3 shows a similar spatial orientation that may lead to a similar effect preventing the fibril or oligomers from further growth. Notably, this sequence motif was absent in the PHF6*-binding peptides MMD3 and MMD3rev reported in Malhis et al. [52].

Our NMR data show that ISAD1 does bind Tau monomers in solution only moderately. Since the ELISA was carried out with high local concentrations of Tau that could lead to oligomerization, it is likely that Tau was immobilized as a mixture of monomeric and oligomeric forms, and the affinity measured by ELISA reflects the binding of ISAD1 to this mixture. The NMR spectra also support the claim that ISAD1 promotes the formation of high molecular weight oligomers of Tau. The reference Tau^FL^ spectrum contained a mixture of monomeric and oligomeric Tau, oligomer formation being favored in the absence of reducing agents. No marked increase in *I*/*I*_o_ values was evident and there are generally many *I*/*I*_o_ values below 1 especially for the mole ratio 1:30 Tau:ISAD1 (Sup. Fig. 2B). This suggests that at high concentrations of ISAD1 the affinity of ISAD1 for monomeric Tau is moderate, and Tau oligomer formation is favored in the presence of ISAD1.

Aggregation of Tau is the primary hallmark for the disease pathology in AD and other tauopathies. A significant inhibition of the aggregation by therapeutic molecules is considered beneficial. Our novel peptide ISAD1 inhibited the fibrillization of Tau^FL^ and pro-aggregant Tau^RDΔK^ (IC_50_ of ~3 μM) in concentrations which are typical for peptide inhibitors. Interestingly, ISAD1 inhibited fibrillization of Tau more efficiently than other PHF6 addressing peptides: TLKIVW 54.1 μM [9] and APT 5.9 μM [50].

Familial mutations of Tau cause the rapid aggregation and progression of the diseases. This makes it necessary to find a D-peptide that can inhibit the aggregation of such pro-aggregant mutants of Tau. To date, only our D-peptides have been proven to simultaneously inhibit the aggregation of three pro-aggregant Tau forms (Tau^FLΔK^, Tau^FL-A152T^, Tau^FL-P301L^) found in FTD, AD, PSP, and FTDP-17 diseases (Fig. 5A–C) in a concentration-dependent manner which makes our D-peptides unique and proves their therapeutic potential. Mutations ΔK280 and P301L are each located on R2 of the repeat domain, close to a possible binding site of ISAD1 while mutation A152T is located in the proline-rich domain within Tau. The inhibitory potencies are almost the same for Tau^FL^ and Tau^RDΔK^ (IC_50_ of 2.9 μM), despite the fact that the two Tau constructs have different aggregation efficiencies.

Based on DLS and pelleting assays, we observed that the ISAD1 and its reversed form prevent fiber formation of Tau^FL^ and Tau^RDΔK^ and instead induce the formation of higher molecular weight off-pathway Tau oligomers (Fig. 6B) which are non-fibrillar in nature (ThS negative), similar to other previously described peptides [43, 52]. We had demonstrated by atomic force microscopy (AFM) that in case of the MMD3 peptide, the aggregates formed are amorphous clumps of off-pathway high-n oligomers [52] which cause large signals in DLS experiments, also observed for the ISAD1and its reversed form.

In order to be used as a therapeutic agent for neurodegenerative diseases, the D-peptides need to cross the blood-brain barrier (BBB), should be actively taken up by neurons, and should be non-toxic to brain cells. Peptides do in general not cross membranes very well, but the naturally occurring transcription factor domain penetratin, HIV-Tat, or synthetic cationic peptides have been described as cell penetrating peptides [75–77].

Interestingly, D-peptides investigated previously have also been shown to cross the BBB in combination with high bioavailability and drug exposure to the brain [45]. The novel D-peptides developed in this study were demonstrated to cross the membranes of N2a-Tau^RDΔK^ cells efficiently (Fig. 7), although the mechanism of penetration is still unclear. Since the N2a-Tau^RDΔK^ cells take up the peptides uniformly, we assume that the uptake of the peptides occurs through bulk endocytosis. Our D-peptides neither caused a change in cell viability (Fig. 8A) nor cell membrane integrity (Fig. 8B) even at high concentrations, suggesting high tolerability by neuronal cells. Additionally, ISAD1 and its reversed form prevented the cytotoxic potential of Tau aggregates by promoting off-pathway high-n oligomers, evident from the enhanced cell viability (Fig. 9A) in N2a-Tau^RDΔK^ cells, improved cell membrane integrity (Fig. 9B), and prevent the ROS elevation (Fig. 9C). Compared to other Tau-derived peptides previously published [78–80], ISAD1 demonstrates similar prevention of toxicity by maintaining cell viability and metabolic activity also in the presence of cellular Tau^RDΔK^ expression. The N2a-Tau^RDΔK^ cell model of Tau pathology is well established and useful in the screening and study of therapeutic compounds, such as Tau aggregation inhibitors [81, 82]. Earlier studies with N2a-Tau^RDΔK^ cells had shown a time-dependent increase in cell death [81]. In N2a-Tau^RDΔK^ cells, the expression of Tau^RDΔK^ starts at 24 h after protein induction and its overexpression is the trigger for dimerization and aggregation. The treatment of N2a-Tau^RDΔK^ cells with ISAD1 and its reversed form decreased the toxicity of cellular Tau^RDΔK^ in a dose-dependent manner, as seen by the parameters of cell viability and LDH release (Fig. 10). This demonstrates that toxic effects of Tau^RDΔK^ aggregates can be suppressed by the D-peptides.

In conclusion, especially our novel D-amino acid peptide ISAD1 inhibits fibril formation of pro-aggregant toxic Tau, is non-toxic to cells, and prevents the toxic effects of Tau by promoting off-pathway aggregate formation which makes our D-peptide a potential therapeutic molecule to prevent Tau pathology in AD and other Tau-associated diseases. However, ISAD1rev might have a limited therapeutic potential due to significant lower inhibition of Tau aggregation. More details like BBB transfer and the efficacy of ISAD1 have to be investigated in future in vivo treatment studies to further elucidate the peptide mechanism of action and its full therapeutic potential.

### Limitations

This report has some limitations. The study resulted in very promising findings in vitro and in cell culture; however, BBB permeability and efficiency in vivo still need to be investigated.

## Conclusion

Taken together, using phage display with immobilized Tau^FL^, we have found novel D-amino acid peptides, which inhibited the fibrillization of Tau^FL^. One of our selected D-amino acid peptides, ISAD1, showed the most promising characteristics in several in vitro experiments. We found that ISAD1 was able to bind to Tau^FL^ regardless of its conformation. PHF6 was identified as a possible binding site of ISAD1, binding here in the same mode as already known PHF6 targeting peptides. ISAD1 modulated Tau fibrillization towards nontoxic, non-fibrillar aggregates and therefore rescued cells from Tau-derived toxicity. Additionally, our D-amino acid peptides inhibited the fibrillization of disease-relevant Tau mutants which gives them an extended field of application. The D-amino acid conformation will enable high proteolytic stability of the peptides in vivo. Further investigations including in vivo studies should provide more information about the mechanism how ISAD1 modulates Tau, and should investigate BBB transfer to elaborate the full therapeutic potential.

## Supplementary Information

**Additional file 1.** Supplementing Data.

## Data Availability

All relevant data are within the paper and its Supporting Information files. Additional datasets during and/or analyzed during the current study are available from the corresponding author on reasonable request.

## Abbreviations

AA: Amino acids
AD: Alzheimer’s disease
AFM: Atomic force microscopy
Aβ: Amyloid-beta
BBB: Blood-brain barrier
BCA: Bicinchoninic acid
BSA: Bovine serum albumin
D3: Aβ-binding peptide
Da: Dalton
DCF: Dichlorodihydrofluorescein
DLS: Dynamic light scattering
Dox: Doxycycline
DTT: Dithiothreitol
ELISA: Enzyme-linked immunosorbent assay
FAM: Fluorescein amidites
FBS: Fetal bovine serum
FDA: Food and Drug Administration
FITC: Fluorescein isothiocyanate
FTDP-17: Parkinsonism linked to chromosome 17
G418: Geneticin
H16K: Heparin 16000
HRP: Horseradish peroxidase
Hyg: Hygromycin
LDH: Lactate dehydrogenase
MEM: Minimum essential medium
MT: Microtubules
N2a: Neuronal-2a cell line
NFTs: Neurofibrillary tangles
PBS: Phosphate-buffered saline
PBST: Phosphate-buffered saline with Tween20
PHFs: Paired helical filaments
PSP: Progressive supra-nuclear palsy
PVDF: Polyvinylidene fluoride
RD: Repeat domain
RD2: Peptide derivate of D3
ROS: Reactive oxygen species
RT: Room temperature
SDS-PAGE: Sodium dodecylsulfate polyacrylamide gel electrophoresis
Tau^FL^: Full-length human Tau (441 AA)
Tau^FLΔK^: Full-length Tau mutant ΔK280
Tau^FL-A152T^: Full-length Tau mutant A152T
Tau^FL-P301L^: Full-length Tau mutant P301L
Tau^RDΔK^: Pro-aggregant repeat domain Tau mutant ΔK280
TBHP: Tert-butylhydroperoxide
TBS: Tris-buffered saline
TBST: Tris-buffered saline with Tween20
ThT: Thioflavin-T
ThS: Thioflavin-S
TMB: Tetramethylbenzidine
TUP: Target-unrelated peptide
UT: Untreated
