# Supplementary material for: A novel D-amino acid peptide with therapeutic potential (ISAD1) inhibits aggregation of neurotoxic disease-relevant mutant Tau and prevents Tau toxicity in vitro
Source: Alzheimers Res Ther. 2022 Jan 21;14:15. doi: 10.1186/s13195-022-00959-z (PMC8783508; doi:10.1186/s13195-022-00959-z)
Supplement: Supplementary file 1 — Additional file 1. Supplementing Data. [file 13195_2022_959_MOESM1_ESM.docx]

# **Supplementing Data Aillaud et al.**

**Peptide synthesis of ISAL5 to ISAL8 and ISAL1sam**

Peptides were synthesized as C-terminal amides by Fmoc/t-Bu-based solid-phase synthesis, as previously described [1]. For ISAL1sam, carbofluorescein was coupled to the ε-amino group of a C-terminally added lysine residue, which was Alloc-protected. A spacer amino acid (8-amino-3,6-dioxaoctanoic acid) was inserted between the peptide sequence and the fluoresceinylated lysine. The Alloc group was selectively removed by overnight treatment with a solution of Pd(PPh_3_)_4_, (8.6 mg/ml) and 1,3-dimethylbarbituric acid (13 mg/ml) in DMF under argon. Crude peptides were purified by preparative HPLC, and purified peptides were then characterized by analytical HPLC with online ESI mass spectrometry detection (LC-MS). Stock solutions of purified peptides were prepared at 2.5 mM in 50 % acetonitrile/water.


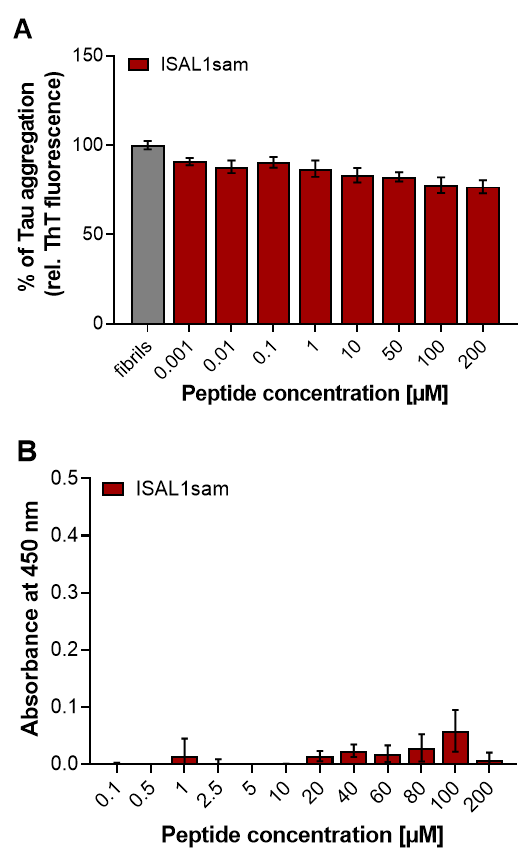


**S****.-Figure 1: ISAL1sam does not inhibit fibril formation of Tau^FL^ and shows no binding to Tau^FL^ in ELISA. (A)** To monitor fibril formation, 5 µM of Tau^FL^ was diluted with 1.25 µM heparin (16000 Da) and 10 µM ThT in HEPES buffer. The peptide ISAD1sam was added in different concentrations (0.001 to 200 µM) and incubated at 37 °C for 48 hours. The fluorescence of the respective Tau fibril control after 48 hours was set as 100 % and the values and standard deviations of the other incubations are given as percentages of this maximum value. **(B)** Tau^FL^ was immobilized at a concentration of 5 µg/ml and incubated with 0.1 – 200 µM of the FAM-labelled peptide ISAL1sam. Detection was performed using an HRP-coupled anti-FITC antibody (1:5000). The data represent the means ± SD with n = 3 determinations after subtraction of the background signal (wells without Tau).

**NMR titration experiments with Tau^FL^**

ISAD1 peptide stock solutions (10 mM) were prepared in Tris-buffered saline (TBS, 50 mM Tris, 150 mM NaCl, pH 7.6). ^15^N-labeled Tau^FL^ protein (Uniprot ID 10636-8, 441 residues) was prepared using a previously described method [2]. To observe the effect of ISAD1 on backbone amides of Tau^FL^, NMR titrations were performed following the procedure we previously described for the MMD3 peptide [3]. In such titrations, 2D ^1^H-^15^N SOFAST-Heteronuclear Multiple Quantum Coherence (HMQC) [4] spectra of ^15^N-labeled Tau^FL^ (18 µM) were collected with Tau^FL^:peptide mole ratios of 1:3, 1:10, and 1:30. Spectra were acquired at 5 °C on a Bruker 800 MHz spectrometer equipped with a triple-resonance cryoprobe. NMR titration samples were prepared in 50 mM sodium phosphate buffer at pH 6.8, 0.01 % NaN_3_, 90 % H_2_O/10 % D_2_O, and were incubated for about 16 hours at 37 °C prior to recording SOFAST-HMQC spectra. In control experiments, reference samples containing Tau^FL^ in the above mentioned phosphate buffer were titrated with TBS. NMR spectra were processed using TopSpin version 3.6.2 (Bruker) and analyzed using Sparky [5]. For each titration point, NMR signal intensity ratios (I/I_0_ ) and chemical shift perturbations (CSP) were determined. The signal intensity ratio is determined by dividing the intensity (I) of Tau^FL^ amide peaks in the presence of ISAD1 by the corresponding peak intensity (I_0_ ) in the absence of ISAD1. CSPs were calculated using the equation CSP = {√ [0.5 [(ΔδH)^2^ + (ΔδN)^2^/25]}, where ΔδH and ΔδN are the chemical shift differences of H and N taken from Tau^FL^ spectra in the presence and absence of ISAD1.


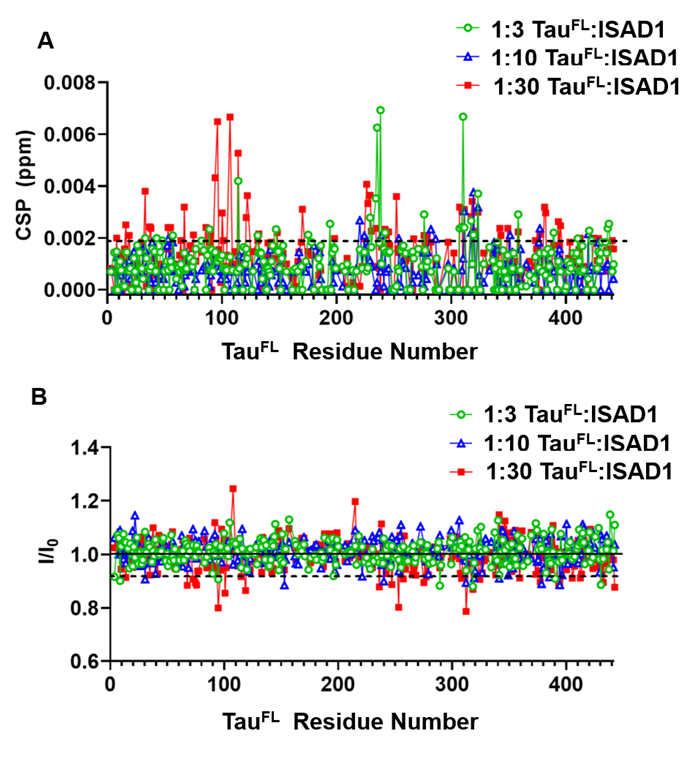


**S.-Figure 2: NMR spectroscopy of the interaction between ISAD1 and Tau^FL^**

**(A)** CSPs induced by the addition of ISAD1 to Tau^FL^ at Tau^FL^:ISAD1 mole ratios 1:3 (green), 1:10 (blue), and 1:30 (red). The dotted black line represents a cut-off of 0.0020 ppm, which is two standard deviations above the average CSP value for the mole ratio 1:10 **(B)** NMR signal intensity ratios I/I_0_ from 2D ^1^H-^15^N SOFAST-HMQC spectra corresponding to Tau^FL^:ISAD1 mole ratios 1:3 (green), 1:10 (blue), and 1:30 (red). The dotted black line corresponds to a threshold of 0.92, which is two standard deviations below the average I/I_0_ value for the mole ratio 1:10.


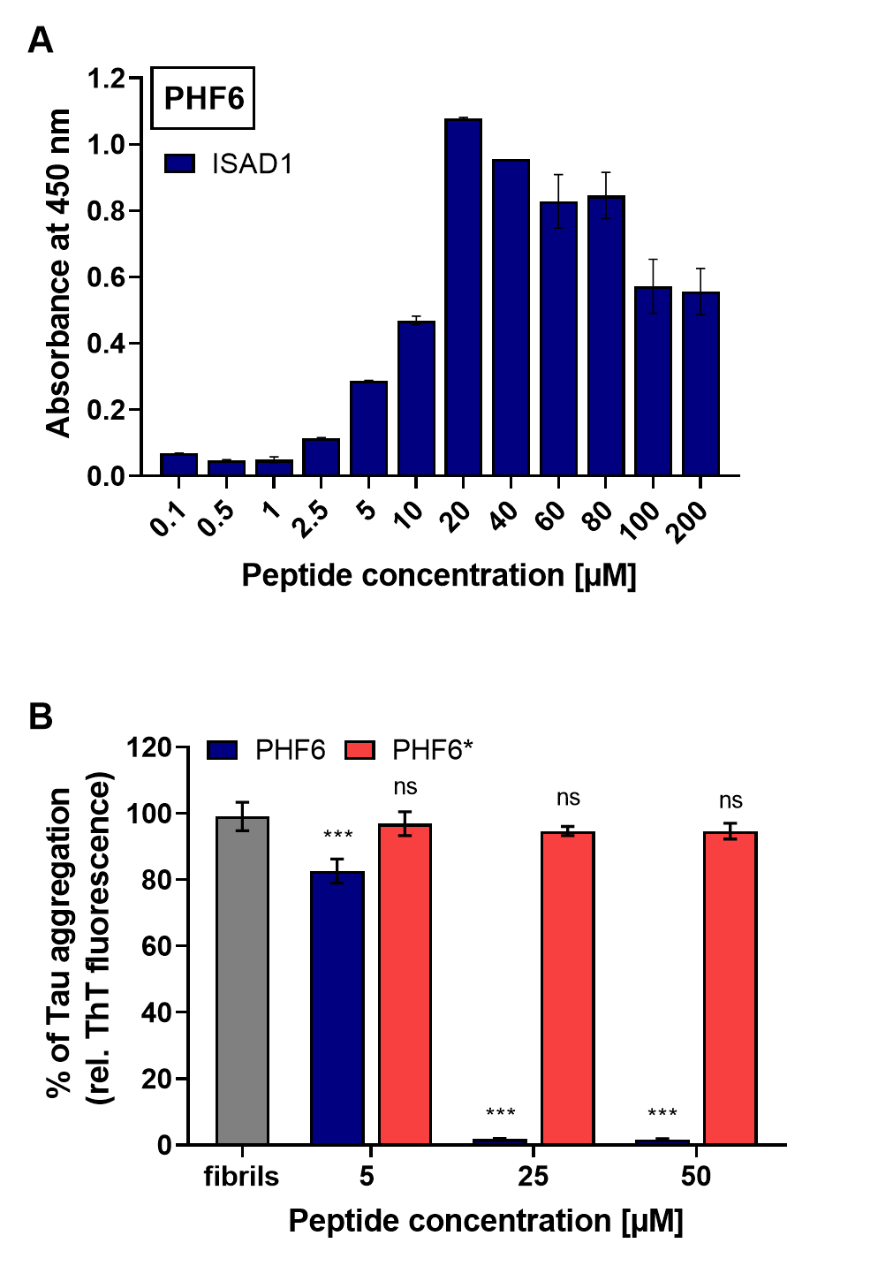


**S.-Figure 3: ISAD1 shows binding to PHF6 in ELISA and** **inhibits fibril formation of PHF6, while fibril formation of PHF6* was not inhibited. (A)** Binding properties of ISAD1 to PHF6. The plate was coated with 5 µg/ml of PHF6 and blocked with 3 % BSA. As negative control, only coating buffer was used instead of the hexapeptide. The FAM-labeled peptides were added in different concentrations from 0.1 µM to 200 µM. For the detection of bound peptides, a horseradish peroxidase-conjugated sheep anti-FITC antibody was used. The absorption of the triplicates was measured at 450 nm. **(B)** PHF6 and PHF6*, respectively, (5 μM) were co-incubated (24 hours) with different concentrations of ISAD1 (5, 25 and 50 µM). The relative fluorescence of a buffer sample was subtracted. Fluorescence was measured at 520 nm in relative units after 24 hours and set as 100 %. The data represent the mean values ± SD with three replicates per run. Significant differences are shown in relation to the fibrils control (grey) [n = 3; one-way ANOVA with Tukey´s post hoc test; *F*(6, 56) = 2455; ***p ≤ 0.001].


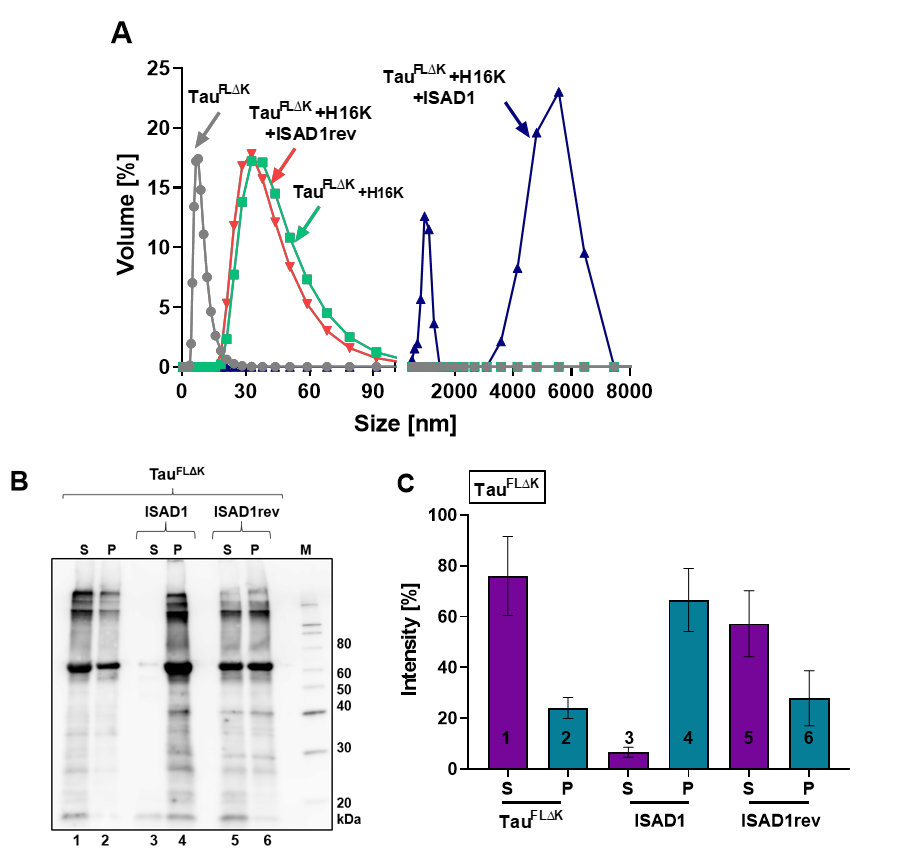


**S-Figure 4-6: ISAD1 induces the formation of non-fibrillar** **large aggregates of Tau mutants. (A)** 10 µM Tau^FLΔK^ (Sup. Fig. 2) or Tau^FL-A152T^ (Sup. Fig. 3) or Tau^FL-P103L^ (Sup. Fig. 4) along with 2.5 µM heparin and 20 µM ThS was incubated in the presence and absence of D-peptides (100 µM concentration) for 24 hours. The aggregated material was used for DLS measurements. The average of 3 measurements within 15 runs is shown as volume graph. Tau^FLΔK^ (Sup. Fig. 2A), Tau^FL-A152T^ (Sup. Fig. 3A) or Tau^FL-P301L^ (Sup. Fig. 4A) monomers show a hydrodynamic size of < 10 nm diameter (grey curve). When Tau is incubated with heparin, larger fibrils with a diameter between 10 and 100 nm are formed (green curve). Aggregates which are formed by addition of ISAD1rev are comparable to those formed by incubation with heparin (red curve). Only in the presence of ISAD1, even larger aggregates are formed, with a diameter of 500 - 8000 nm (blue curve). **(B)** Western blot analysis of the aggregated samples of Tau^FL-ΔK^ (Sup. Fig. 2), Tau^FL-A152T^ (Sup. Fig. 3) or Tau^FL-P301L^ (Sup. Fig. 4). After incubation (24 hours) the aggregated samples were centrifuged to separate the supernatant (S) form the pellet (P). Detection was performed by the antibody K9Ja using chemiluminescence method. Tau^FLΔK^ (Sup. Fig. 2B), Tau^FL-A152T^ (Sup. Fig. 3B) or Tau^FL-P301L^ (Sup. Fig. 4B) fibrils samples, show soluble Tau (fractions in supernatant) and insoluble Tau (pellet) (lanes 1,2). Samples with ISAD1rev show more fractions in the supernatant (lanes 5,6) similar to the heparin induced fibrils. ISAD1 causes the formation of high molecular weight aggregates which are only visible in the pellet (lane 4). **(C)** The intensity of protein amount in supernatant and pellet was quantified by ImageJ for different Tau mutants such as Tau^FLΔK^ (Sup. Fig. 2C), Tau^FL-A152T^ (Sup. Fig. 3C) or Tau^FL-P301L^ (Sup. Fig. 4C). The results are comparable to those of the western blot.


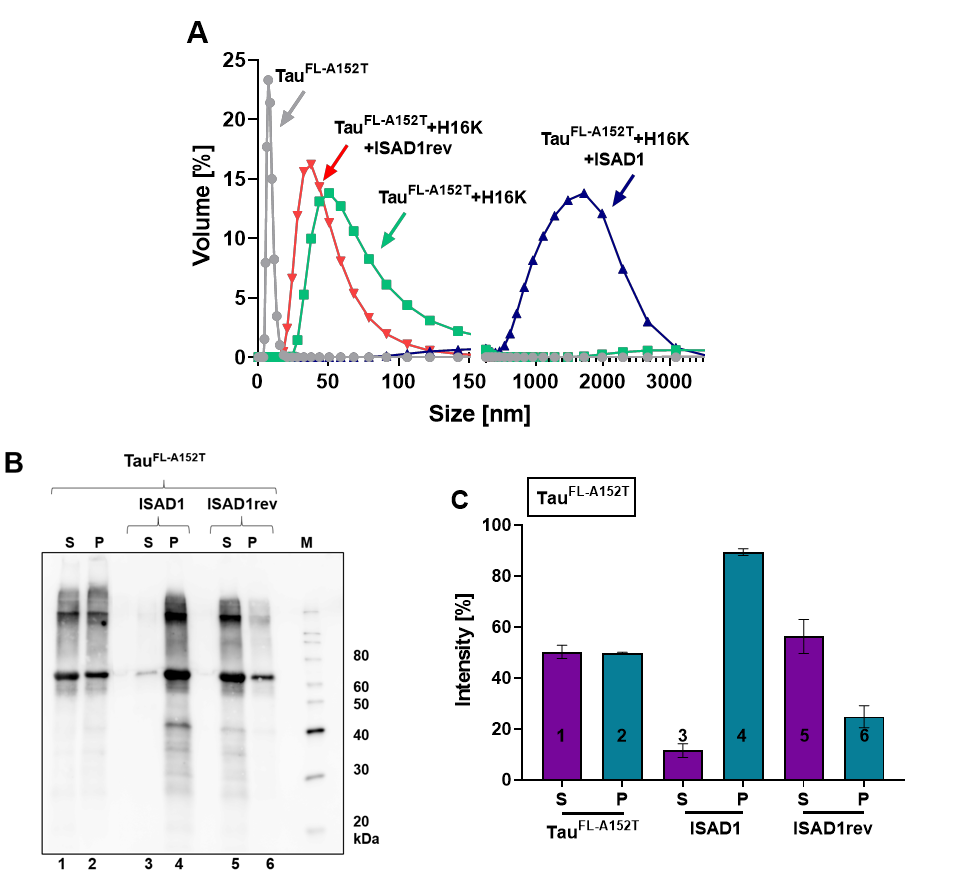


**S-Figure 5: ISAD1 induces the formation of non-fibrillar large aggregates of Tau mutants.**


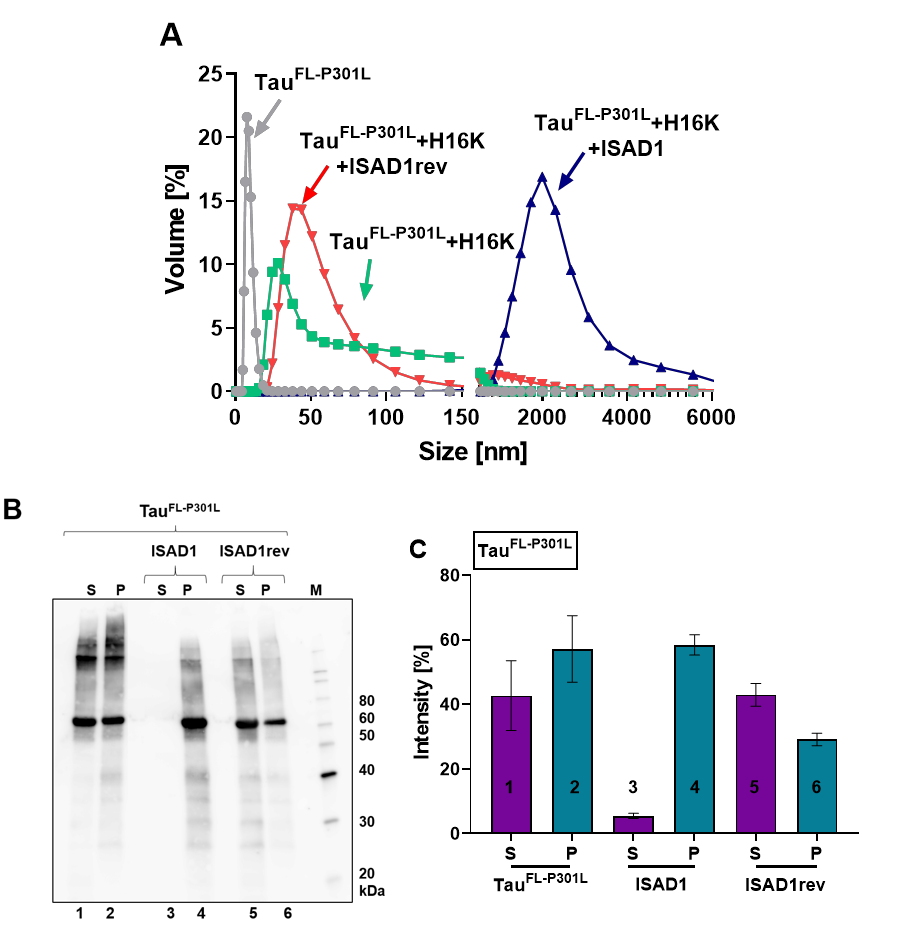


**S-Figure 6: ISAD1 induces the formation of non-fibrillar large aggregates of Tau mutants.**


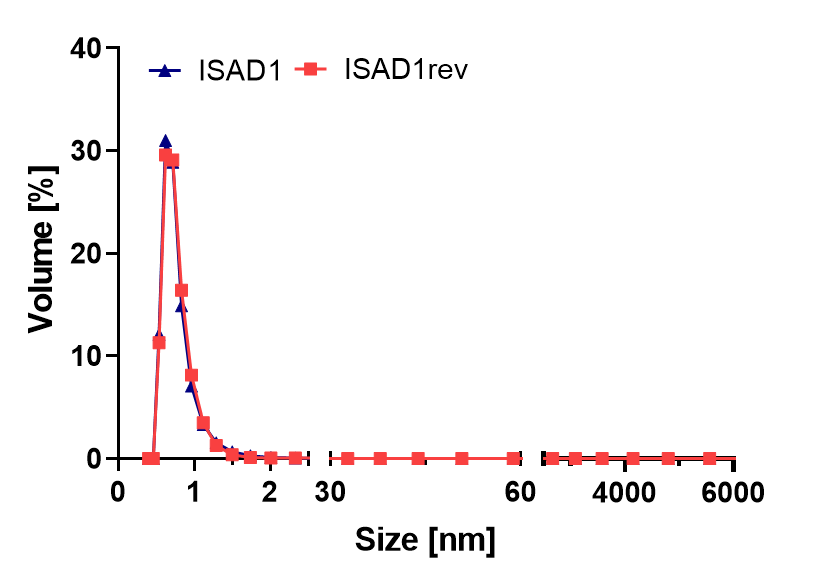


**S-Figure 7: ISAD1 and ISAD1rev do not form larger aggregates theirselves.** 100 μM of D-peptides and 2.5 µM of heparin 16000 (H16K) were incubated together for 24 hours and measured by DLS. Particles of ISAD1 peptide (blue curve) and ISAD1rev (red curve) are < 2 nm in size which confirm that peptides alone in the presence of heparin do not form larger aggregates.

References

1. Groß A, Rödel K, Kneidl B, Donhauser N, Mössl M, Lump E, et al. Enhancement and induction of HIV-1 infection through an assembled peptide derived from the CD4 binding site of gp120. Chembiochem. 2015;16:446–54. doi:10.1002/cbic.201402545.

2. Ukmar-Godec T, Fang P, Ibáñez de Opakua A, Henneberg F, Godec A, Pan K-T, et al. Proteasomal degradation of the intrinsically disordered protein tau at single-residue resolution. Sci Adv. 2020;6:eaba3916. doi:10.1126/sciadv.aba3916.

3. Malhis M, Kaniyappan S, Aillaud I, Chandupatla RR, Ramirez LM, Zweckstetter M, et al. Potent Tau Aggregation Inhibitor D-Peptides Selected against Tau-Repeat 2 Using Mirror Image Phage Display. ChemBioChem 2021. doi:10.1002/cbic.202100287.

4. Schanda P, Kupce E, Brutscher B. SOFAST-HMQC experiments for recording two-dimensional heteronuclear correlation spectra of proteins within a few seconds. J Biomol NMR. 2005;33:199–211. doi:10.1007/s10858-005-4425-x.

5. Lee W, Tonelli M, Markley JL. NMRFAM-SPARKY: enhanced software for biomolecular NMR spectroscopy. Bioinformatics. 2015;31:1325–7. doi:10.1093/bioinformatics/btu830.
